# Supplementary material for: Phytoplankton thermal responses adapt in the absence of hard thermodynamic constraints
Source: Evolution. 2020 Mar 13;74(4):775–90. doi: 10.1111/evo.13946 (PMC7384082; doi:10.1111/evo.13946)
Supplement: Supplementary file 1 — Figure S1: Three alternative ways in which a weak hotter‐is‐better pattern may emerge. Figure S2: Isolation locations of phytoplankton species, coloured by phylum. Figure S3: The ratio of B(T ref) to B pk for experimentally‐determined TPCs with a growth rate value of 0 s−1 at the rising part. Figure S4: Estimates obtained with a T ref value of 0°, for phytoplankton species/strains available in our phylogeny and with known isolation locations. Figure S5: Estimates obtained with a T ref value of 10°C, and excluding fits with T pk < 10°C, for phytoplankton species/strains available in our phylogeny and with known isolation locations. Figure S6: Violin plots of Effective Sample Size (ESS) and Potential Scale Reduction Factor (PSRF) values for the parameters of the evolutionary model across all ExaBayes runs. Figure S7: Pairwise comparisons of split frequencies among the four ExaBayes runs, indicating excellent convergence. Figure S8: Projection of the sampled tree topologies in two dimensions for each ExaBayes run. Figure S9: Violin plots of Effective Sample Size (ESS) and Potential Scale Reduction Factor (PSRF) values for the parameters of the evolutionary model across all ExaBayes runs. Figure S10: Pairwise comparisons of split frequencies among the four ExaBayes runs, indicating excellent convergence. Figure S11: Projection of the sampled tree topologies in two dimensions for each ExaBayes run. Figure S12: Heat map of Matching Cluster distances between all produced tree topologies and that of the Open Tree of Life (v. 4). Figure S13: Statistical support – in terms of bootstrap values or posterior probabilities – for the nodes of the extended RAxML topology, according to RAxML, PhyML, and ExaBayes. Figure S14: Violin plots of Effective Sample Size (ESS) and Potential Scale Reduction Factor (PSRF) values for the parameters of the relative time‐calibration model across the five DPPDiv runs. Figure S15: The phylogeny of phytoplankton species in this study, with branches [file EVO-74-775-s001.pdf]

Phytoplankton thermal responses adapt in the absence of hard thermodynamic constraints.

Dimitrios - Georgios Kontopoulos[1,2,*], Erik van Sebille[3,4], Michael Lange[5], Gabriel Yvon-Durocher[6], Timothy G. Barraclough[2], and Samraat Pawar[2]

Supporting Information

Contents

S1 Variants of the weak hotter-is-better hypothesis **2**

S2 Isolation locations of species/strains in this study **2**

S3 Estimation of TPC parameter values **3**
S3.1 Derivation of the Sharpe-Schoolfield equation with T_{pk} as a parameter 3
S3.2 Non-positive growth rate values and the Sharpe-Schoolfield model 4
S3.3 Fitting the Sharpe-Schoolfield model to growth rate data 5

S4 Phylogeny reconstruction diagnostics and tree comparisons **7**
S4.1 Convergence diagnostics for ExaBayes (only required species) 7
S4.2 Convergence diagnostics for ExaBayes (with extra species) 9
S4.3 Comparison of resulting phylogenetic topologies 11
S4.4 Convergence diagnostics for the relative time-calibration (DPPDiv) 13

S5 Multi-response Markov chain Monte Carlo generalised linear mixed models **14**
S5.1 Using the entire dataset 14
S5.1.1 Variance/covariance matrices with T_{ref} set to 0°C 18
S5.1.2 Variance/covariance matrices with T_{ref} set to 10°C 20
S5.2 Using the marine subset of the dataset 22
S5.2.1 With T_{ref} set to 0°C 22
S5.2.2 With T_{ref} set to 10°C 27

S6 Scaling of B_0 and B_{pk} with cell volume **33**
S6.1 Regressions with B_0 and B_{pk} appropriately transformed towards normality 33
S6.2 Regressions with log-transformed B_0 33

S7 List of 16S/18S rRNA sequences used for phylogeny reconstruction **35**

---

1. Science and Solutions for a Changing Planet DTP. **2.** Department of Life Sciences, Imperial College London, Silwood Park, Ascot, Berkshire SL5 7PY, UK. **3.** Grantham Institute, Imperial College London, London SW7 2AZ, UK. **4.** Institute for Marine and Atmospheric Research Utrecht, Utrecht University, Utrecht 3584 CC, the Netherlands. **5.** Department of Earth Science and Engineering, Imperial College London, London SW7 2AZ, UK. **6.** Environment and Sustainability Institute, University of Exeter, Penryn, Cornwall TR10 9EZ, UK. * Corresponding author; e-mail: d.kontopoulos13@imperial.ac.uk.

S1 Variants of the weak hotter-is-better hypothesis

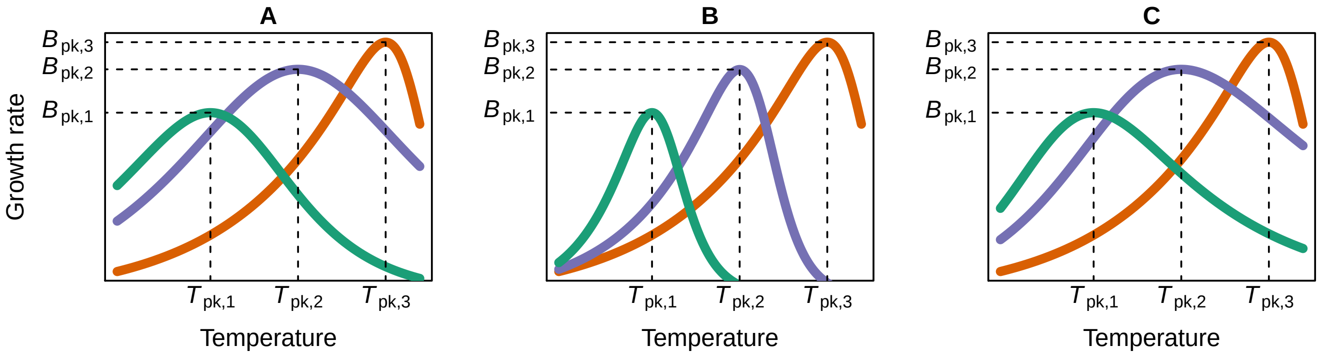

Figure S1: Three alternative ways in which a weak hotter-is-better pattern may emerge. A: The slope of the rising part of the TPC (E) remains fixed, whereas the baseline height (B_0) varies across species. B: B_0 is fixed, whereas E varies. C: Both B_0 and E exhibit variation. The variant that is closest to the expectations of the Metabolic Theory of Ecology is that of panel A. To facilitate the comparison of the three variants of weak hotter-is-better, we kept the same B_{pk} and T_{pk} values across the three panels.

S2 Isolation locations of species/strains in this study

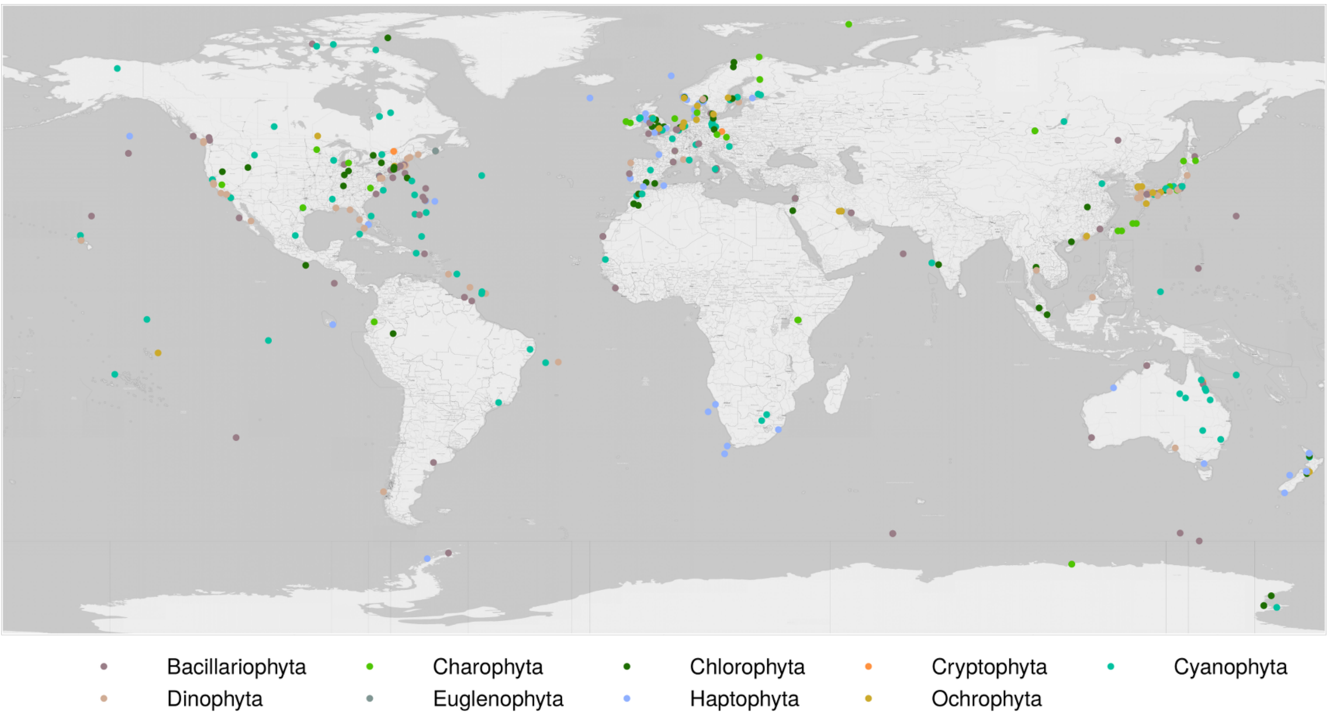

Figure S2: Isolation locations of phytoplankton species, coloured by phylum.

S3 Estimation of TPC parameter values

S3.1 Derivation of the Sharpe-Schoolfield equation with T_{pk} as a parameter

The original equation of the four-parameter Sharpe-Schoolfield model (Schoolfield et al., 1981) includes the parameter T_{h}, which stands for the temperature at which half of the population of the key rate-limiting enzyme is made inactive:

$$B(T) = B_0 \cdot \frac{e^{\left[\frac{-E}{k}\cdot\left(\frac{1}{T}-\frac{1}{T_{\mathrm{ref}}}\right)\right]}}{1+e^{\left[\frac{E_{\mathrm{D}}}{k}\cdot\left(\frac{1}{T_{\mathrm{h}}}-\frac{1}{T}\right)\right]}}. \tag{S1}$$

As T_{h} can be lower or higher than the temperature at which the thermal performance curve peaks (T_{pk}), there is not a straightforward mapping between the value of T_{h} and the effect of temperature on the shape of the thermal performance curve. T_{pk} is a more useful parameter from a physiological standpoint and, as we had previously shown in Kontopoulos et al. (2018), it can be analytically estimated as

$$T_{\mathrm{pk}} = \frac{-E_{\mathrm{D}} \cdot T_{\mathrm{h}}}{k \cdot T_{\mathrm{h}} \cdot \ln \frac{E}{E_{\mathrm{D}}-E} - E_{\mathrm{D}}}. \tag{S2}$$

Then, to reformulate Eq. (S1) with T_{pk}, we first solve Eq. (S2) for T_{h}:

$$T_{\mathrm{pk}} = \frac{-E_{\mathrm{D}} \cdot T_{\mathrm{h}}}{k \cdot T_{\mathrm{h}} \cdot \ln \frac{E}{E_{\mathrm{D}}-E} - E_{\mathrm{D}}} \implies T_{\mathrm{pk}} \cdot \left(k \cdot T_{\mathrm{h}} \cdot \ln \frac{E}{E_{\mathrm{D}}-E} - E_{\mathrm{D}}\right) = -E_{\mathrm{D}} \cdot T_{\mathrm{h}} \implies$$

$$\implies T_{\mathrm{h}} \cdot \left(k \cdot T_{\mathrm{pk}} \cdot \ln \frac{E}{E_{\mathrm{D}}-E} + E_{\mathrm{D}}\right) = E_{\mathrm{D}} \cdot T_{\mathrm{pk}} \implies T_{\mathrm{h}} = \frac{E_{\mathrm{D}} \cdot T_{\mathrm{pk}}}{k \cdot T_{\mathrm{pk}} \cdot \ln \frac{E}{E_{\mathrm{D}}-E} + E_{\mathrm{D}}}.$$

Finally, we substitute T_{h} with the above quantity in Eq. (S1):

$$B(T) = B_0 \cdot \frac{e^{\left[\frac{-E}{k}\cdot\left(\frac{1}{T}-\frac{1}{T_{\mathrm{ref}}}\right)\right]}}{1+e^{\left[\frac{E_{\mathrm{D}}}{k}\cdot\left(\frac{1}{T_{\mathrm{h}}}-\frac{1}{T}\right)\right]}} = B_0 \cdot \frac{e^{\left[\frac{-E}{k}\cdot\left(\frac{1}{T}-\frac{1}{T_{\mathrm{ref}}}\right)\right]}}{1+e^{\left[\frac{E_{\mathrm{D}}}{k}\cdot\left(\frac{k \cdot T_{\mathrm{pk}} \cdot \ln \frac{E}{E_{\mathrm{D}}-E} + E_{\mathrm{D}}}{E_{\mathrm{D}} \cdot T_{\mathrm{pk}}}-\frac{1}{T}\right)\right]}} =$$

$$= B_0 \cdot \frac{e^{\left[\frac{-E}{k}\cdot\left(\frac{1}{T}-\frac{1}{T_{\mathrm{ref}}}\right)\right]}}{1+e^{\left[\ln \frac{E}{E_{\mathrm{D}}-E}\right]} \cdot e^{\left[\frac{E_{\mathrm{D}}}{k}\cdot\left(\frac{1}{T_{\mathrm{pk}}}-\frac{1}{T}\right)\right]}} = B_0 \cdot \frac{e^{\left[\frac{-E}{k}\cdot\left(\frac{1}{T}-\frac{1}{T_{\mathrm{ref}}}\right)\right]}}{1+\frac{E}{E_{\mathrm{D}}-E} \cdot e^{\left[\frac{E_{\mathrm{D}}}{k}\cdot\left(\frac{1}{T_{\mathrm{pk}}}-\frac{1}{T}\right)\right]}}.$$

S3.2 Non-positive growth rate values and the Sharpe-Schoolfield model

The Sharpe-Schoolfield model (Eq. (1) in the main text) has an exponential (Arrhenius) term in its numerator and, as a result, the model cannot predict non-positive rate values. Thus, before fitting the model to data, we removed any growth rate measurements ≤ 0 s^{-1}. The only model parameter whose estimate will not be biologically realistic in such a case is B_0 (the rate performance at a low reference temperature), which can only take positive values. To assess the impact of this issue on our results, we examined the number of non-positive growth rate measurements at the rise of the TPC (which is partly controlled by B_0). Across all species in our final TPC dataset, very few species were exclusively represented by TPCs with negative growth rate values at the rising part (less than 2%). The corresponding number for TPCs that had zero values at the rise was 19%.

For the latter group of TPCs, we also calculated the ratio of rate performance at $T_{\text{ref}} = 0°\text{C}$ to the maximum rate performance (B_{pk}). The numerator of the ratio (and the ratio itself) should be 0, as in these TPCs, there was at least one zero growth rate measurement at a temperature above 0°C. Indeed, as shown in Fig. S3, the ratio is very close to zero in most cases. This indicates that B_0 estimates are, in general, only slight overestimates of true growth rate values and that they do not systematically bias the results of our study.

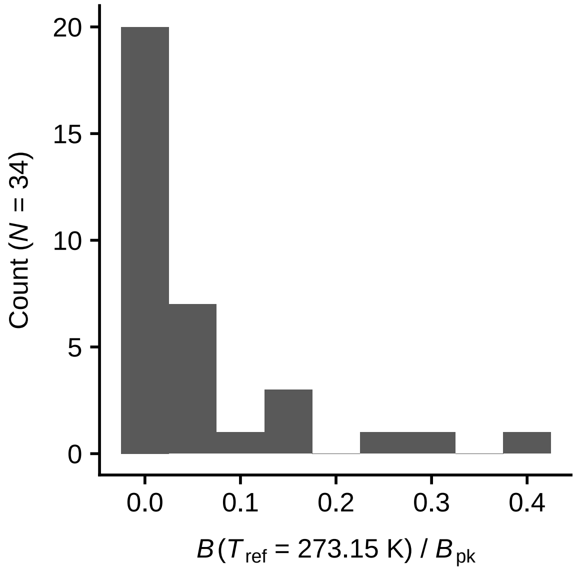

Figure S3: The ratio of $B(T_{\text{ref}})$ to B_{pk} for experimentally-determined TPCs with a growth rate value of 0 s^{-1} at the rising part. Most values are close to the true ratio of 0.

S3.3 Fitting the Sharpe-Schoolfield model to growth rate data

To facilitate nonlinear least squares fitting, we set bounds to three parameters of the model (Table S1). Also, during the optimization process, when the value of E was greater than E_{D}, we forced the objective function to return 10^{10} instead of the expected rate value. This strict penalization forced the nonlinear least squares optimizer to accept only values of $E < E_{\mathrm{D}}$, in line with experimentally observed thermal performance curves. Additionally, we enforced extremely strict convergence criteria for the optimization, by setting the tolerance parameters xtol and ftol to the value of 10^{-12}, and the maximum number of iterations to 100,000.

**Parameter**	**Lower bound**	**Upper bound**
E	10^{-5}	10
E_{D}	10^{-5}	30
T_{pk}	263.15	423.15

Table S1: Parameter bounds set for nonlinear least squares fitting.

We fitted the model to each species or strain whose growth rate had been measured at a minimum of five different temperatures. After fitting the model, we filtered the fits to ensure only reliable and useful parameter estimates – within the context of this study – were retained. To this end, we first rejected i) fits with $R^2 < 0.5$, ii) E estimates that were unrealistically high (i.e., greater than 4 eV), iii) fits where $T_{\mathrm{pk}} < T_{\mathrm{ref}}$ (in the case of $T_{\mathrm{ref}} = 10°\mathrm{C}$), iv) parameter estimates from species that were not in our phylogeny, and v) parameter estimates from species whose isolation location was not available. Next, we ensured that there was an adequate number of data points at each area of the thermal performance curve for the resulting parameter estimates to be reasonably accurate (see Pawar et al. 2016). More precisely, we rejected B_0 and E estimates if there were fewer than four data points below T_{pk}. T_{pk} and B_{pk} estimates were accepted if there were at least two data points both before and after T_{pk}. E_{D} values were rejected if there were fewer than four data points after T_{pk}. Finally, to accept W_{op} estimates, we required four data points before T_{pk} and two after it.

The distributions of the accepted parameter estimates are shown in Figures S4 ($T_{\mathrm{ref}} = 0°\mathrm{C}$) and S5 ($T_{\mathrm{ref}} = 10°\mathrm{C}$).

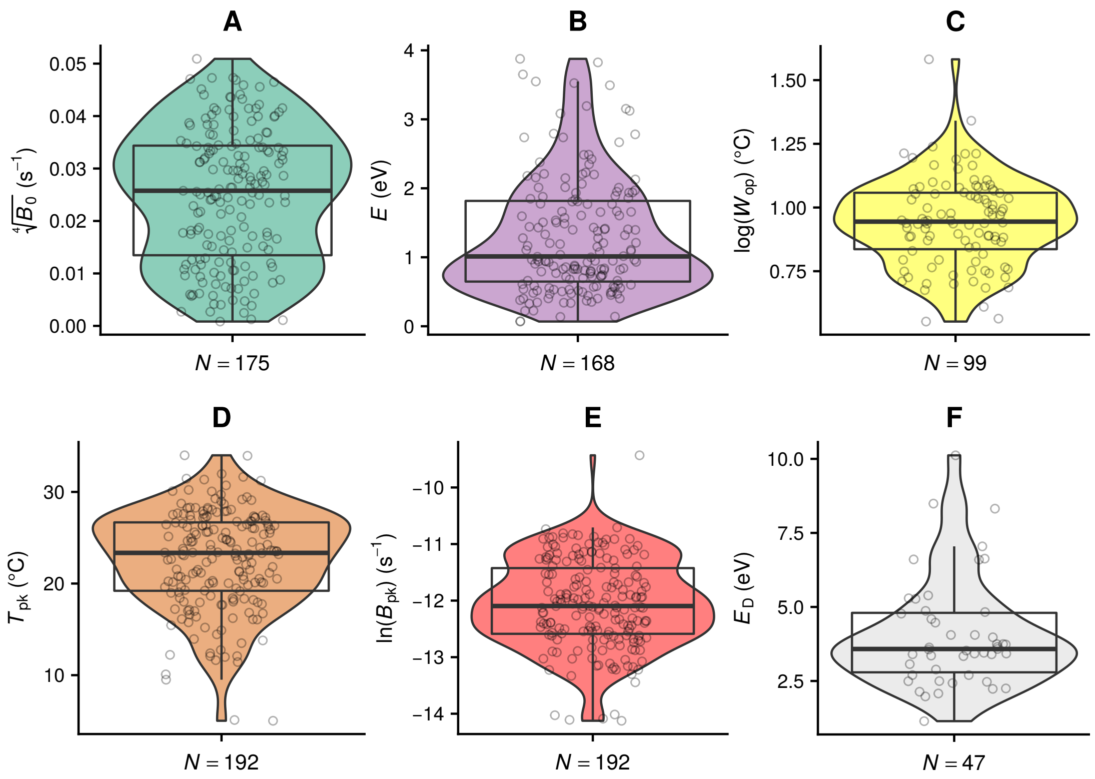

Figure S4: Estimates obtained with a T_{ref} value of 0°C, for phytoplankton species/strains available in our phylogeny and with known isolation locations.

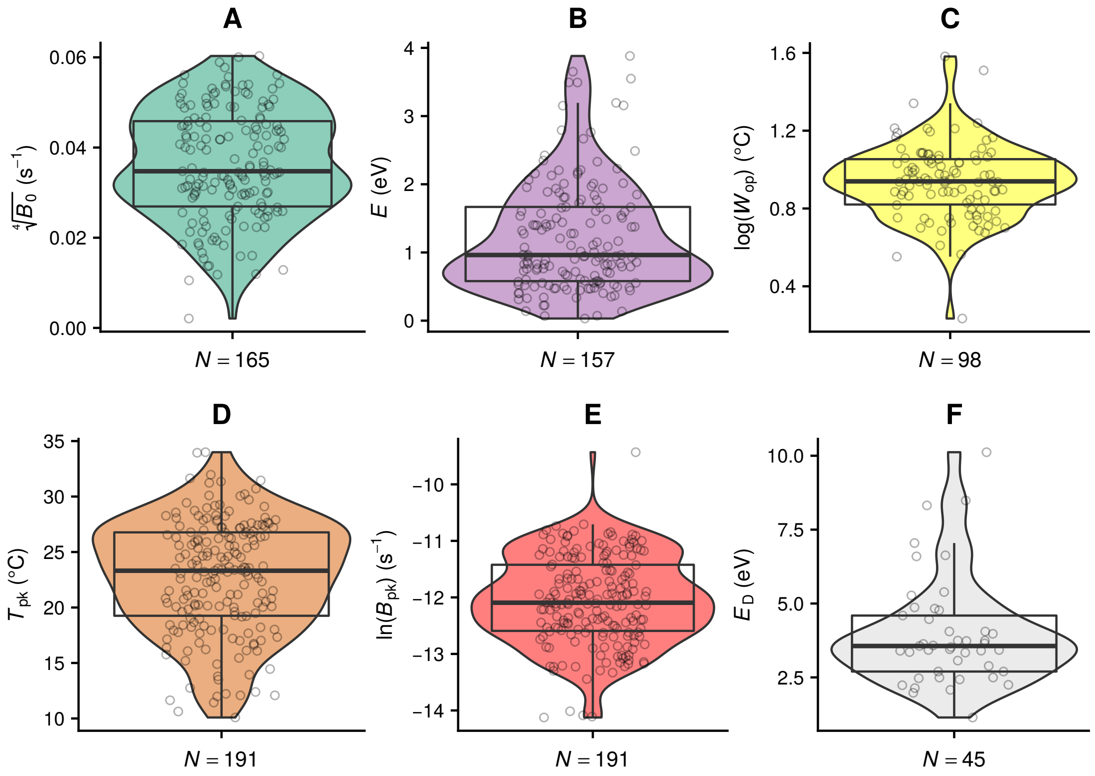

Figure S5: Estimates obtained with a T_{ref} value of 10°C, and excluding fits with $T_{\mathrm{pk}} < 10$°C, for phytoplankton species/strains available in our phylogeny and with known isolation locations.

S4 Phylogeny reconstruction diagnostics and tree comparisons

The next two subsections show the outputs of the diagnostics that we ran to verify that the four independent ExaBayes runs (with or without the addition of extra species) had converged.

S4.1 Convergence diagnostics for ExaBayes (only required species)

The following diagnostic tests were executed with the AWTY web server (Nylander et al., 2007) and the rwty R package (Warren et al., 2017).

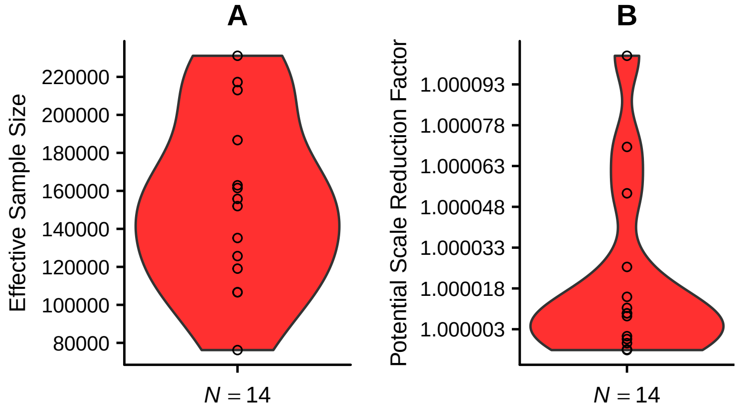

Figure S6: Violin plots of Effective Sample Size (ESS) and Potential Scale Reduction Factor (PSRF) values for the parameters of the evolutionary model across all ExaBayes runs. Convergence and parameter sampling are deemed sufficient if ESS $>$ 200 and PSRF $<$ 1.1.

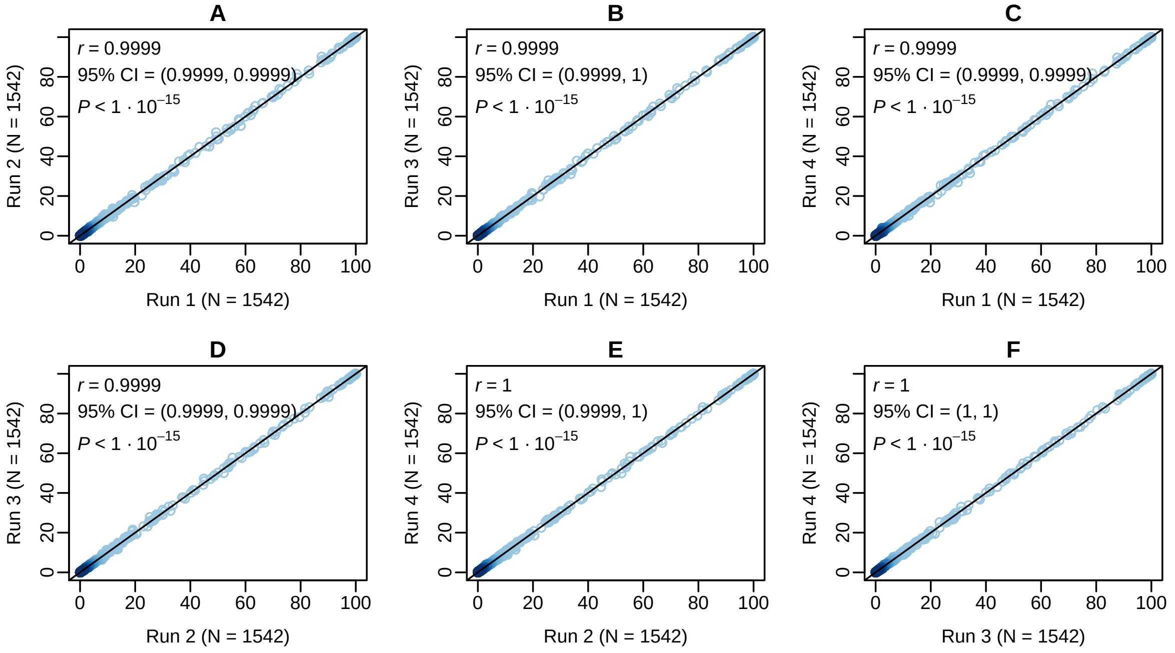

Figure S7: Pairwise comparisons of split frequencies among the four ExaBayes runs, indicating excellent convergence.

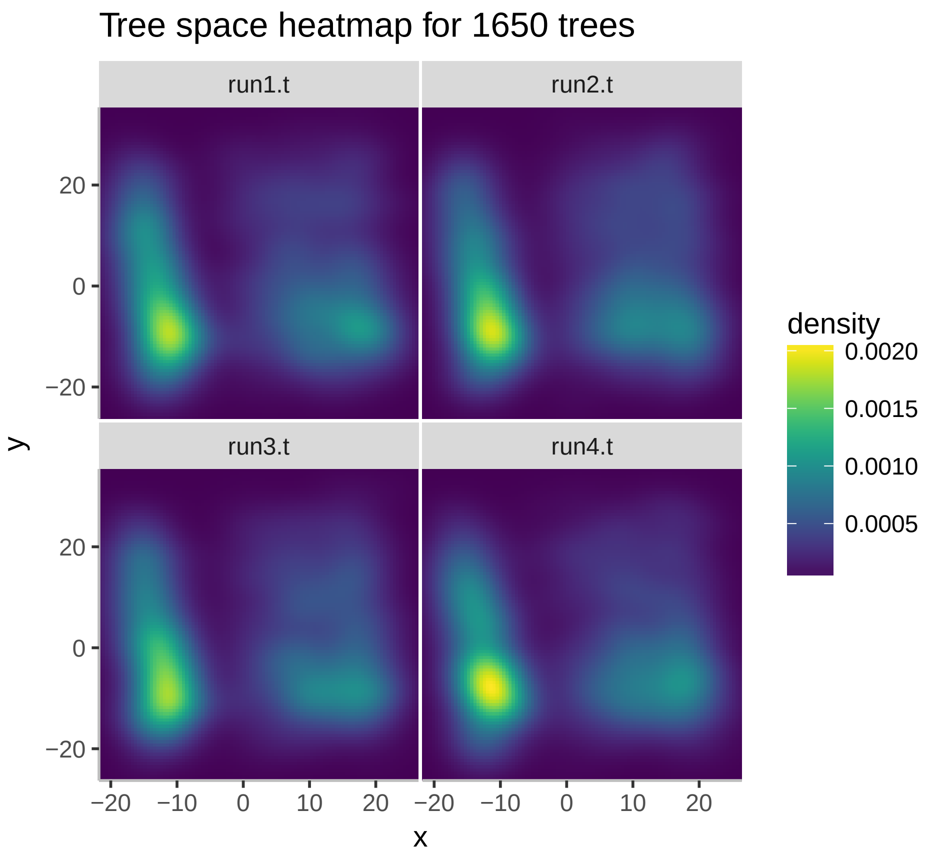

Figure S8: Projection of the sampled tree topologies in two dimensions for each ExaBayes run. As the four panels are highly similar, the runs appear to have adequately converged.

S4.2 Convergence diagnostics for ExaBayes (with extra species)

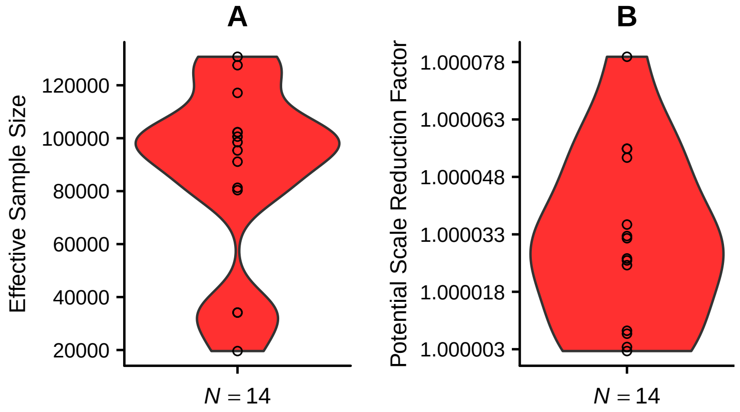

Figure S9: Violin plots of Effective Sample Size (ESS) and Potential Scale Reduction Factor (PSRF) values for the parameters of the evolutionary model across all ExaBayes runs. Convergence and parameter sampling are deemed sufficient if ESS > 200 and PSRF < 1.1.

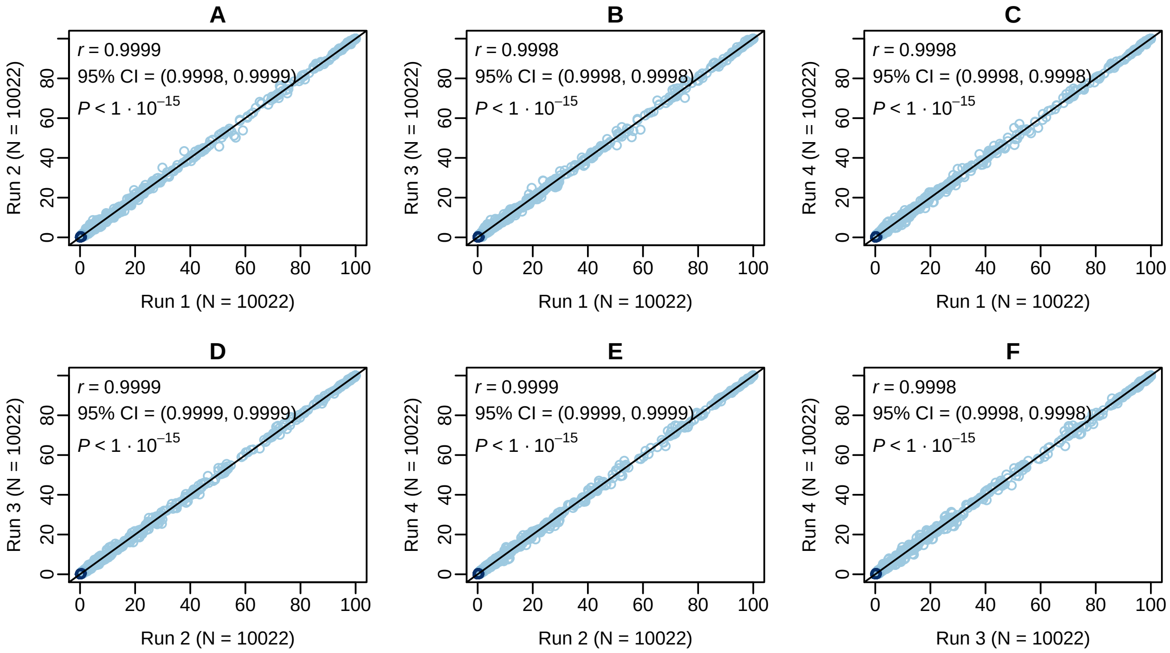

Figure S10: Pairwise comparisons of split frequencies among the four ExaBayes runs, indicating excellent convergence.

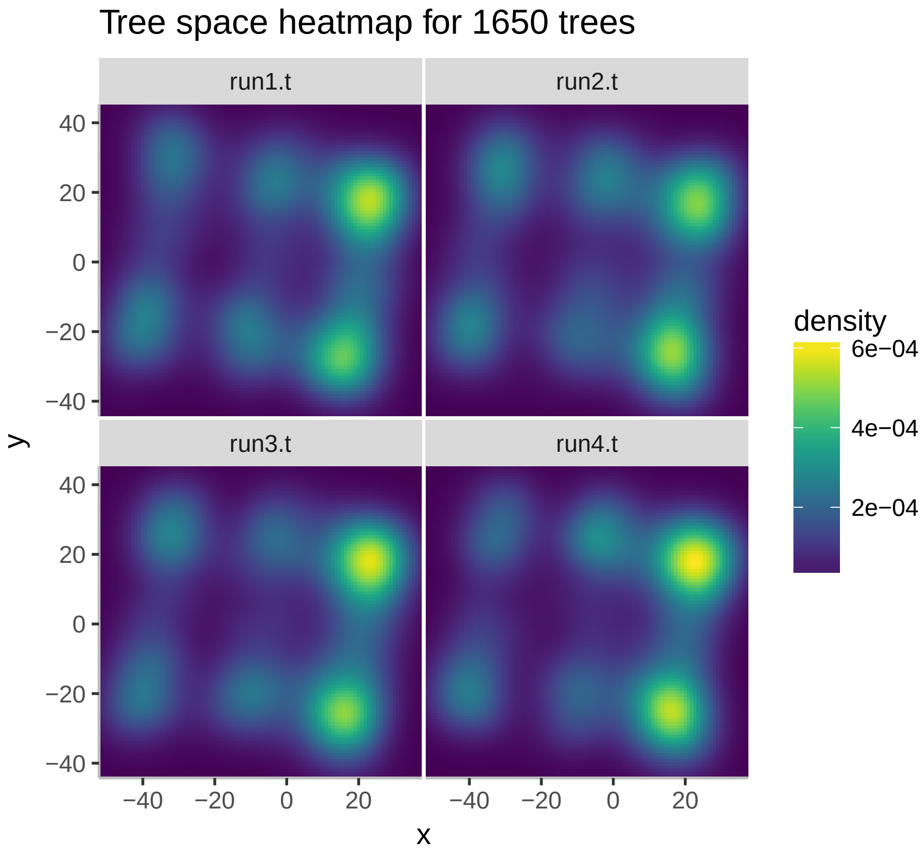

Figure S11: Projection of the sampled tree topologies in two dimensions for each ExaBayes run. As the four panels are highly similar, the runs appear to have adequately converged.

S4.3 Comparison of resulting phylogenetic topologies

To understand whether the addition of extra species improved the tree topology by bringing it closer to our knowledge of the tree of life, we compared all resulting topologies to that of the Open Tree of Life (v. 4; Hinchliff et al. 2015). To this end, we first rooted all trees by setting the prokaryotic Cyanophyta species as an outgroup. We then pruned the trees down to a common subset of species. The Open Tree of Life topology was added in two ways: i) including polytomies (i.e., as originally obtained), and ii) with polytomies resolved by RAxML using our alignment of 16S/18S rRNA sequences. Distances between tree topologies were calculated according to the Matching Cluster metric of Bogdanowicz and Giaro (2013), as implemented in TreeCmp (Bogdanowicz et al., 2012). The topologies that were found closest to the Open Tree of Life were those constructed by ExaBayes and RAxML using the sequence alignment with extra species (Fig. S12).

We then compared the log-likelihood of the ExaBayes and RAxML topologies by evaluating them with RAxML under the GTR+Γ model, which had been used for tree reconstruction. As the RAxML topology had a higher log-likelihood (-109356.08) than that of ExaBayes (-109479.86), we used the RAxML tree for the analyses of this study (Fig. S13).

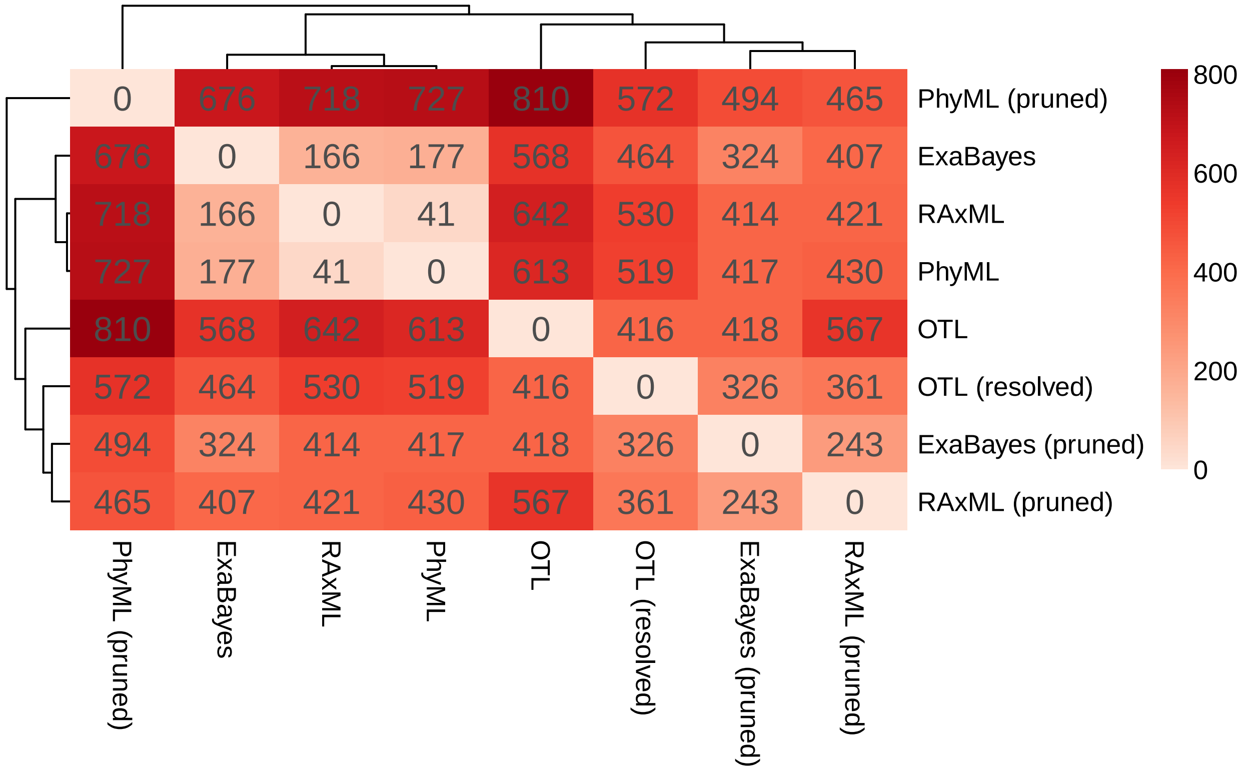

Figure S12: Heat map of Matching Cluster distances between all produced tree topologies and that of the Open Tree of Life (v. 4). Trees marked as "pruned" here are those that included extra species (e.g., macroalgae, land plants). The addition of more species appears to improve the quality of the resulting phylogenetic tree in two of the three programs that we used.

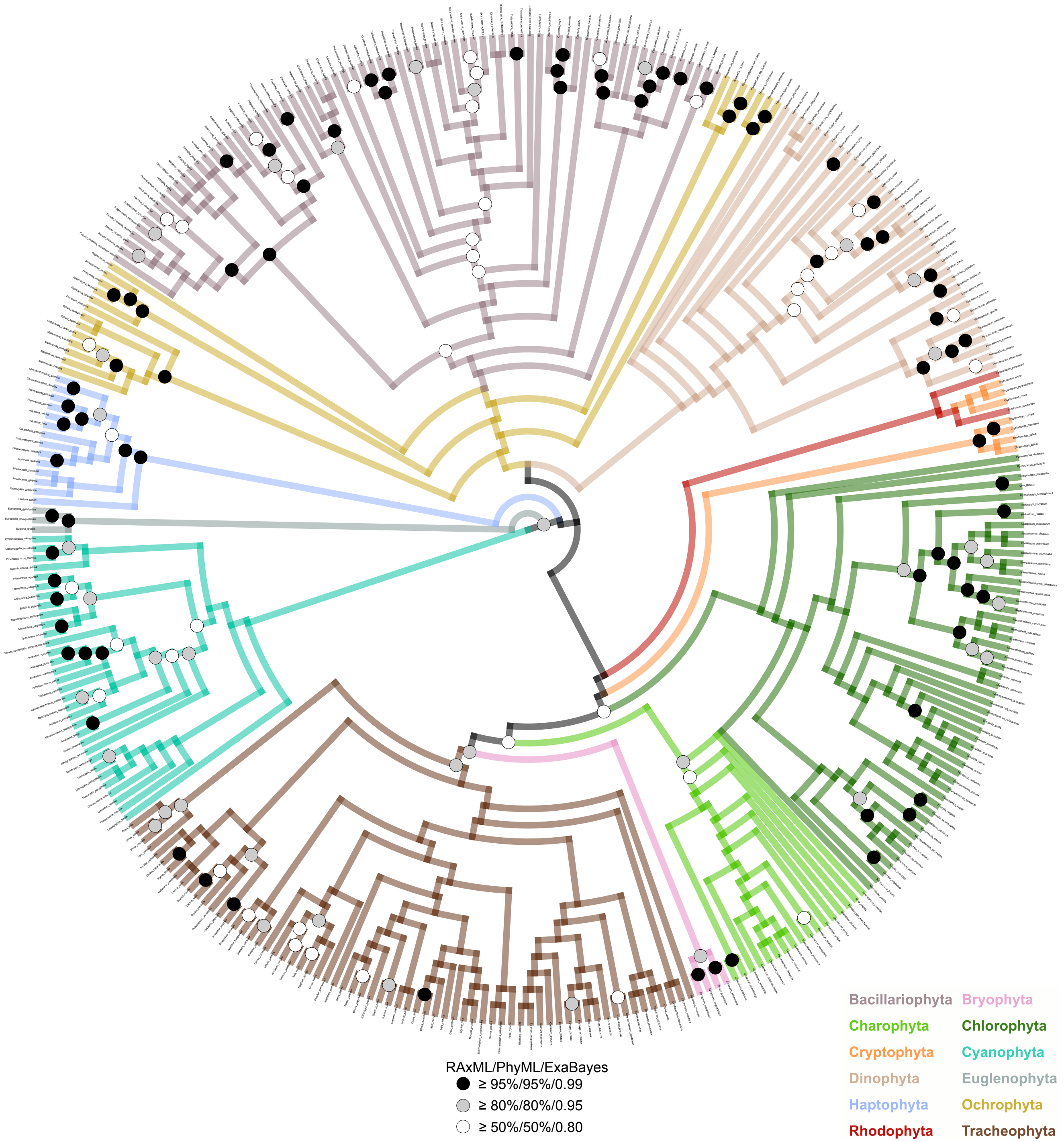

Figure S13: Statistical support – in terms of bootstrap values or posterior probabilities – for the nodes of the extended RAxML topology, according to RAxML, PhyML, and ExaBayes. Nodes with black or grey circles, in particular, are considered robust. Note that, as shown in Fig. S12, the RAxML and ExaBayes topologies are quite close to each other and to the OTL, whereas the PhyML topology is distinctly different. Therefore, the statistical support of many nodes in this figure is probably an underestimate of what we would obtain if we were to exclude the PhyML tree from the comparison.

S4.4 Convergence diagnostics for the relative time-calibration (DPP-Div)

As previously, we performed a diagnostic test to verify that the five independent DPPDiv runs had converged on statistically indistinguishable posterior distributions.

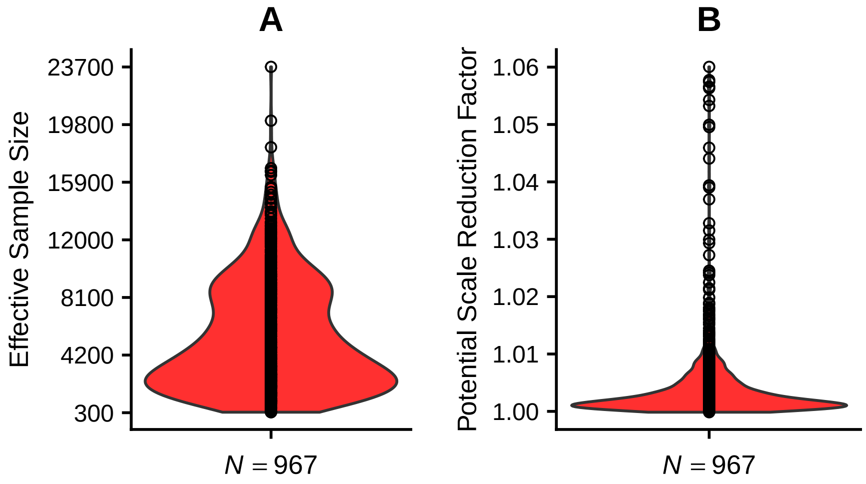

Figure S14: Violin plots of Effective Sample Size (ESS) and Potential Scale Reduction Factor (PSRF) values for the parameters of the relative time-calibration model across the five DPPDiv runs. Convergence and parameter sampling are deemed sufficient if ESS > 200 and PSRF < 1.1.

S5 Multi-response Markov chain Monte Carlo generalised linear mixed models

S5.1 Using the entire dataset

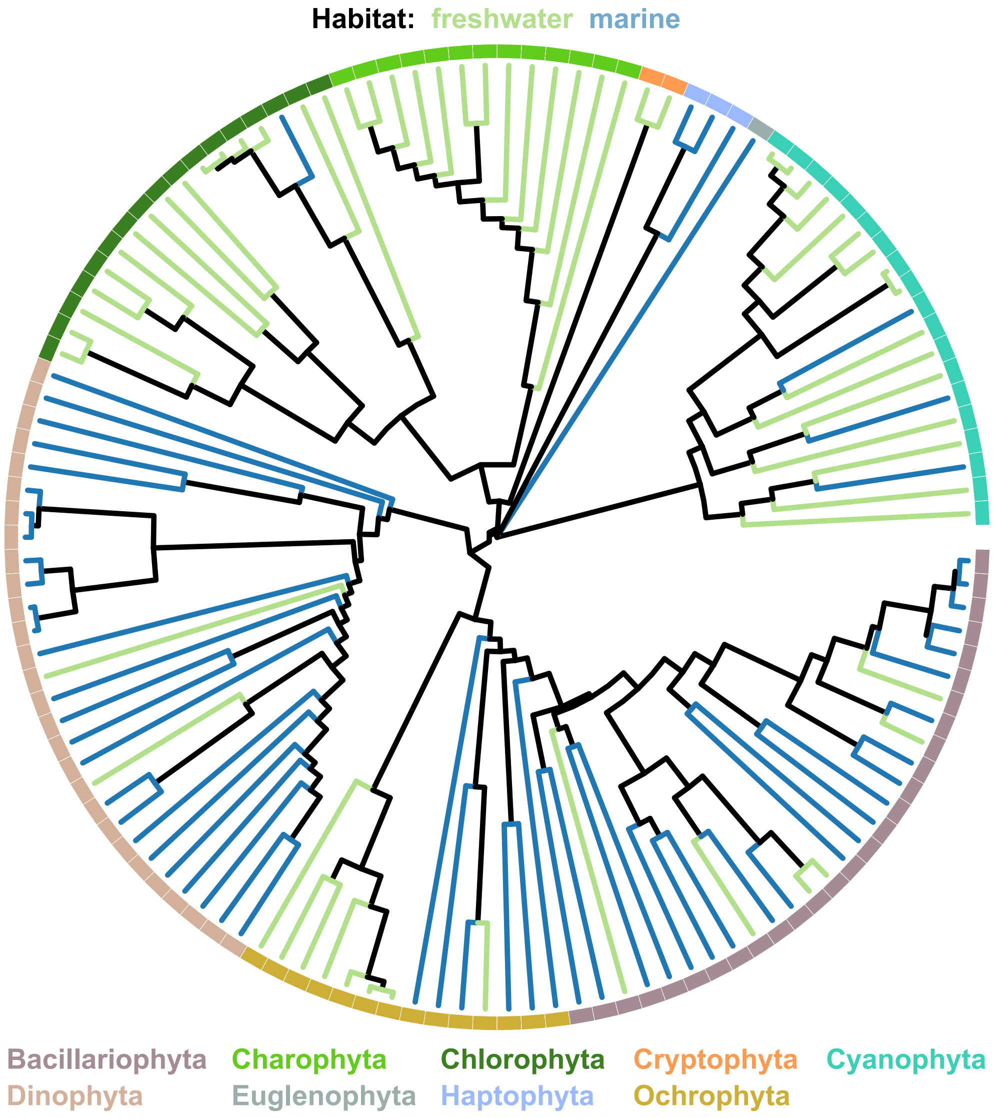

Figure S15: The phylogeny of phytoplankton species in this study, with branches coloured by habitat. The arcs of the circle around the tree denote the various phytoplankton Phyla. This figure was generated using the ggtree R package (Yu et al., 2017).

To assess the extent of phylogenetic sampling bias, we checked whether phylogenetic distance correlates with geographical distance by performing a Mantel test (Mantel, 1967) with 9,999 permutations. In particular, we used the implementation of the Mantel test in the R package ade4 (Dray and Dufour, 2007) to estimate the correlation between the phylogenetic distance matrix and the matrix of geographical distances between species/strains. The latter was calculated using Vincenty's inverse formula (Vincenty, 1975). The correlation between the two matrices was almost nonexistent ($r = 0.0423$, $p = 0.0011$).

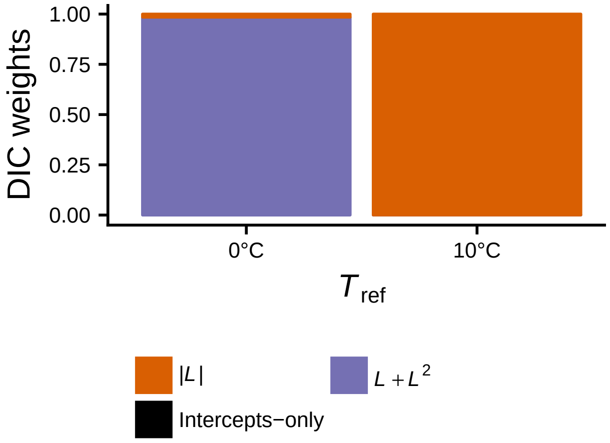

Figure S16: DIC weights of models fitted to the entire dataset of phytoplankton TPC parameters. Models that included the habitat (marine vs freshwater) of the species as a fixed effect are not shown here, as the 95% HPD intervals of the habitat coefficients always contained zero. The best-fitting model had a second-order polynomial in latitude (when T_{ref} was set to 0°C), or absolute latitude (when T_{ref} was set to 10°C) as fixed effects.

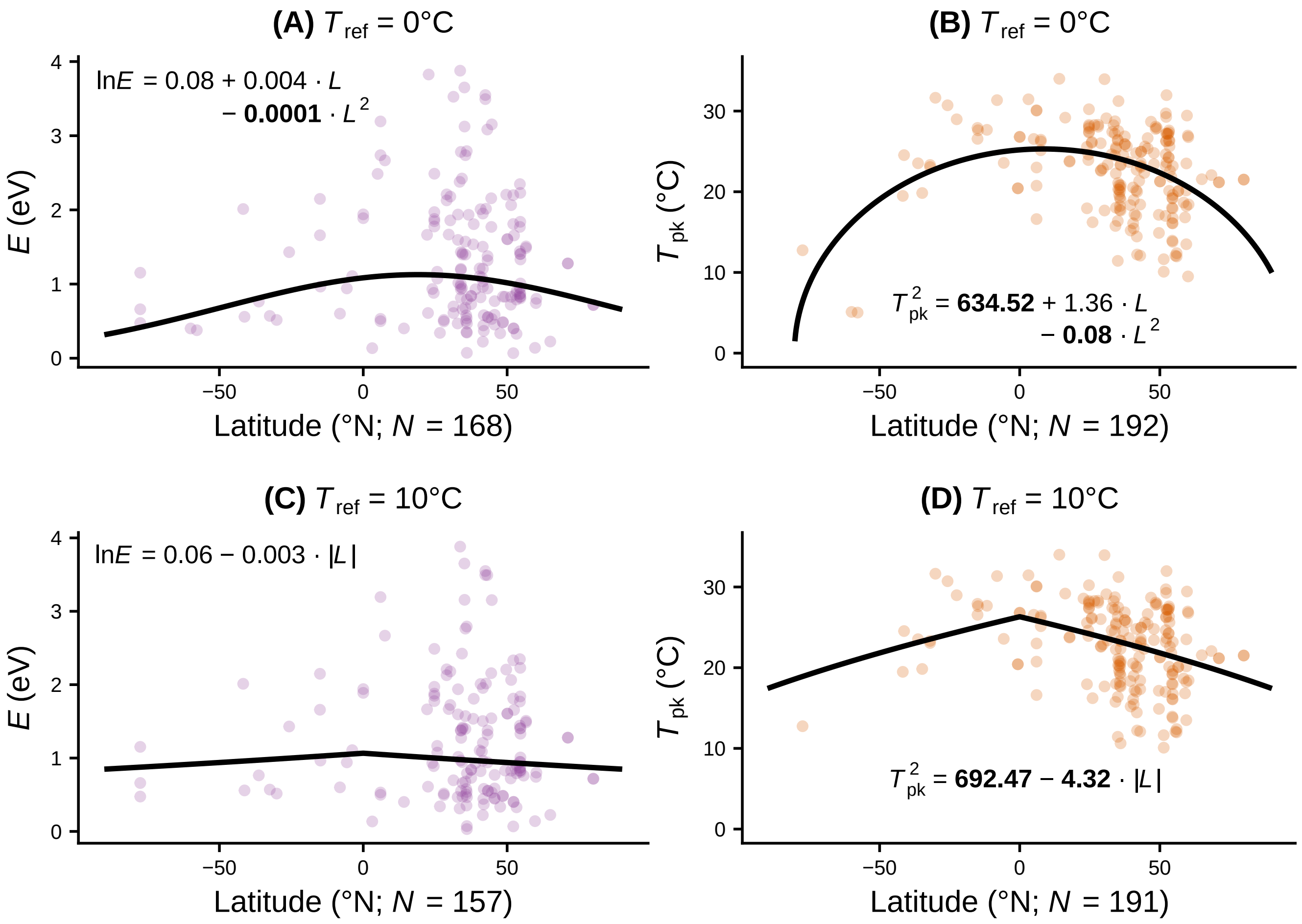

Figure S17: Latitudinal associations of E and T_{pk}. These thermal parameters decrease with absolute latitude, especially if we include species/strains adapted to colder temperatures with $T_{\text{pk}} < 10°\text{C}$. Data points are coloured according to their local density. Coefficients whose 95% Highest Posterior Density interval did not include zero are shown in bold. The lack of the coldest-adapted species changes the best-fitting line from a second-degree polynomial (panels A and B) to a linear relationship with absolute latitude (panels C and D). In panel C, in particular, latitude no longer appears to associate with E, as the 95% Highest Posterior Density interval of its coefficient includes zero. These results suggest that, from the equator to higher latitudes, species adapt by both decreasing their T_{pk} and increasingly becoming thermal generalists.

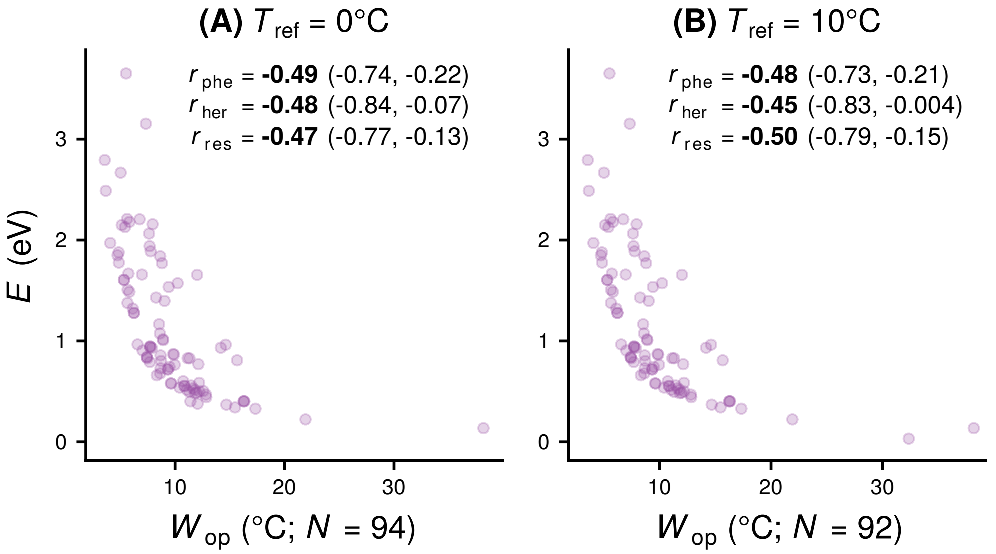

Figure S18: E versus W_{op} using a T_{ref} of 0°C (panel A), or a T_{ref} of 10°C and excluding species/strains with $T_{\mathrm{pk}} < 10$°C (panel B). As expected, there is a negative correlation between the two thermal parameters, as TPCs with very steep rising slopes have a narrow operational niche width, and vice versa. The correlation coefficients were estimated between $\ln(E)$ and $\ln(W_{\mathrm{op}})$. Performing the analysis with a T_{ref} of 0°C (panel A) or 10°C (panel B) led to almost identical estimates.

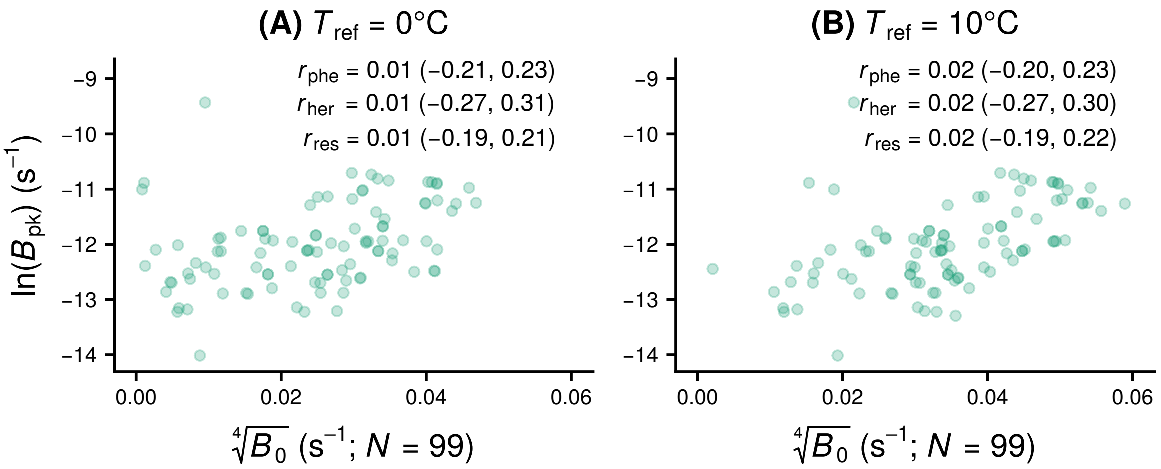

Figure S19: Scatter plots of B_0 at T_{ref} = 0°C (panel A) or T_{ref} = 10°C (panel B). The same sample size across the two panels reflects how a change in the T_{ref} value – when fitting the Sharpe-Schoolfield model – can have a minor impact in the value of R^2, leading to the acceptance of some fitted curves with R^2 values that were previously just below our cutoff of 0.5. While a visual inspection of the plots suggests the presence of a correlation between the two TPC parameters, the correlation becomes non-existent when we control for the phylogenetic distance among species, and the covariance among all TPC parameters. Note that we used different transformations for the two variables in order for their distributions to be as close to normality as possible.

S5.1.1 Variance/covariance matrices with $T_{\mathbf{ref}}$ set to 0°C

	$\sqrt[4]{B_0}$	$\ln(E)$	T_{pk}^2	$\ln(B_{\text{pk}})$	$\ln(E_{\text{D}})$	$\ln(W_{\text{op}})$
$\sqrt[4]{B_0}$	0.022<br>(0.015, 0.030)	-0.003<br>(-0.028, 0.022)	-0.415<br>(-10.422, 9.752)	0.002<br>(-0.034, 0.038)	-0.001<br>(-0.024, 0.022)	0.002<br>(-0.017, 0.021)
$\ln(E)$		0.311<br>(0.102, 0.571)	21.702<br>(-48.743, 95.562)	-0.029<br>(-0.279, 0.215)	0.042<br>(-0.142, 0.242)	-0.119<br>(-0.289, 0.018)
T_{pk}^2			54515.880<br>(29333.040, 83886.920)	75.057<br>(-2.117, 154.759)	25.155<br>(-50.128, 101.150)	-14.416<br>(-73.133, 42.220)
$\ln(B_{\text{pk}})$				0.619<br>(0.320, 0.941)	-0.019<br>(-0.268, 0.232)	0.019<br>(-0.182, 0.223)
$\ln(E_{\text{D}})$					0.274<br>(0.085, 0.517)	-0.029<br>(-0.173, 0.109)
$\ln(W_{\text{op}})$						0.188<br>(0.068, 0.343)

Table S2: Phylogenetically heritable variance/covariance matrix of TPC parameters, as estimated from the intercepts-only model. The first row in each cell stands for the mean value, whereas the second row for the 95% HPD interval.

	$\sqrt[4]{B_0}$	$\ln(E)$	T_{pk}^2	$\ln(B_{\text{pk}})$	$\ln(E_{\text{D}})$	$\ln(W_{\text{op}})$
$\sqrt[4]{B_0}$	0.009<br>(0.007, 0.011)	-0.003<br>(-0.012, 0.007)	0.001<br>(-0.099, 0.100)	0.001<br>(-0.009, 0.010)	0.001<br>(-0.006, 0.007)	0.001<br>(-0.005, 0.007)
$\ln(E)$		0.272<br>(0.174, 0.378)	-0.094<br>(-2.572, 2.356)	-0.014<br>(-0.131, 0.104)	-0.050<br>(-0.152, 0.050)	-0.086<br>(-0.167, -0.008)
T_{pk}^2			44.089<br>(0.067, 158.282)	0.093<br>(-1.869, 2.106)	0.023<br>(-1.357, 1.336)	0.036<br>(-1.216, 1.250)
$\ln(B_{\text{pk}})$				0.227<br>(0.163, 0.296)	0.014<br>(-0.076, 0.103)	-0.008<br>(-0.090, 0.072)
$\ln(E_{\text{D}})$					0.140<br>(0.064, 0.235)	0.011<br>(-0.047, 0.072)
$\ln(W_{\text{op}})$						0.110<br>(0.050, 0.183)

Table S3: Residual variance/covariance matrix of TPC parameters, as estimated from the intercepts-only model. The first row in each cell stands for the mean value, whereas the second row for the 95% HPD interval.

	$\sqrt[4]{B_0}$	$\ln(E)$	T_{pk}^2	$\ln(B_{\text{pk}})$	$\ln(E_{\text{D}})$	$\ln(W_{\text{op}})$
$\sqrt[4]{B_0}$	0.022<br>(0.015, 0.030)	-0.003<br>(-0.029, 0.023)	-0.123<br>(-9.046, 8.602)	0.002<br>(-0.034, 0.039)	0.000<br>(-0.024, 0.022)	0.002<br>(-0.019, 0.021)
$\ln(E)$		0.334<br>(0.111, 0.607)	6.633<br>(-67.701, 81.634)	-0.049<br>(-0.311, 0.212)	0.018<br>(-0.193, 0.244)	-0.132<br>(-0.313, 0.016)
T_{pk}^2			38585.860<br>(19100.750, 61963.260)	66.253<br>(-2.078, 137.745)	16.459<br>(-57.893, 88.398)	-11.019<br>(-68.960, 45.932)
$\ln(B_{\text{pk}})$				0.626<br>(0.326, 0.956)	0.000<br>(-0.274, 0.275)	0.026<br>(-0.189, 0.240)
$\ln(E_{\text{D}})$					0.308<br>(0.092, 0.592)	-0.020<br>(-0.184, 0.138)
$\ln(W_{\text{op}})$						0.204<br>(0.072, 0.373)

Table S4: Phylogenetically heritable variance/covariance matrix of TPC parameters, as estimated from the best fitting model (i.e., with a second order polynomial in latitude as fixed effect).

	$\sqrt[4]{B_0}$	$\ln(E)$	T_{pk}^2	$\ln(B_{\text{pk}})$	$\ln(E_{\text{D}})$	$\ln(W_{\text{op}})$
$\sqrt[4]{B_0}$	0.009<br>(0.007, 0.011)	-0.002<br>(-0.012, 0.007)	0.002<br>(-0.105, 0.107)	0.000<br>(-0.009, 0.010)	0.001<br>(-0.006, 0.007)	0.001<br>(-0.005, 0.007)
$\ln(E)$		0.255<br>(0.161, 0.356)	-0.131<br>(-2.680, 2.342)	-0.005<br>(-0.123, 0.111)	-0.053<br>(-0.153, 0.046)	-0.081<br>(-0.161, -0.007)
T_{pk}^2			56.469<br>(0.069, 188.145)	0.169<br>(-1.868, 2.536)	0.041<br>(-1.497, 1.431)	0.043<br>(-1.302, 1.302)
$\ln(B_{\text{pk}})$				0.230<br>(0.165, 0.300)	0.016<br>(-0.077, 0.110)	-0.010<br>(-0.094, 0.073)
$\ln(E_{\text{D}})$					0.141<br>(0.060, 0.241)	0.014<br>(-0.045, 0.076)
$\ln(W_{\text{op}})$						0.110<br>(0.049, 0.182)

Table S5: Residual variance/covariance matrix of TPC parameters, as estimated from the best fitting model (i.e., with a second order polynomial in latitude as fixed effect).

S5.1.2 Variance/covariance matrices with $T_{\mathbf{ref}}$ set to 10°C

	$\sqrt[4]{B_0}$	$\ln(E)$	T_{pk}^2	$\ln(B_{\text{pk}})$	$\ln(E_{\text{D}})$	$\ln(W_{\text{op}})$
$\sqrt[4]{B_0}$	0.023 (0.015, 0.031)	-0.002 (-0.028, 0.024)	-0.127 (-9.623, 9.479)	0.002 (-0.031, 0.036)	0.000 (-0.024, 0.023)	0.001 (-0.018, 0.021)
$\ln(E)$		0.310 (0.096, 0.588)	-14.496 (-85.719, 57.694)	-0.044 (-0.269, 0.179)	0.016 (-0.173, 0.216)	-0.114 (-0.291, 0.026)
T_{pk}^2			46154.300 (23596.560, 72648.160)	44.455 (-26.466, 117.838)	28.891 (-40.279, 98.378)	-3.217 (-59.759, 53.247)
$\ln(B_{\text{pk}})$				0.536 (0.264, 0.835)	0.023 (-0.217, 0.260)	0.016 (-0.168, 0.200)
$\ln(E_{\text{D}})$					0.269 (0.080, 0.511)	-0.021 (-0.166, 0.116)
$\ln(W_{\text{op}})$						0.187 (0.064, 0.343)

Table S6: Phylogenetically heritable variance/covariance matrix of TPC parameters, as estimated from the intercepts-only model.

	$\sqrt[4]{B_0}$	$\ln(E)$	T_{pk}^2	$\ln(B_{\text{pk}})$	$\ln(E_{\text{D}})$	$\ln(W_{\text{op}})$
$\sqrt[4]{B_0}$	0.010 (0.007, 0.012)	-0.002 (-0.012, 0.009)	0.001 (-0.114, 0.107)	0.001 (-0.009, 0.011)	0.000 (-0.007, 0.008)	0.001 (-0.006, 0.007)
$\ln(E)$		0.294 (0.187, 0.407)	-0.132 (-2.974, 2.582)	-0.022 (-0.145, 0.106)	-0.054 (-0.168, 0.059)	-0.093 (-0.181, -0.008)
T_{pk}^2			52.094 (0.065, 179.992)	0.123 (-2.058, 2.232)	0.021 (-1.477, 1.516)	0.050 (-1.302, 1.404)
$\ln(B_{\text{pk}})$				0.235 (0.170, 0.306)	0.007 (-0.082, 0.097)	-0.003 (-0.088, 0.083)
$\ln(E_{\text{D}})$					0.151 (0.067, 0.253)	0.012 (-0.053, 0.077)
$\ln(W_{\text{op}})$						0.114 (0.051, 0.190)

Table S7: Residual variance/covariance matrix of TPC parameters, as estimated from the intercepts-only model.

	$\sqrt[4]{B_0}$	$\ln(E)$	T_{pk}^2	$\ln(B_{\mathrm{pk}})$	$\ln(E_{\mathrm{D}})$	$\ln(W_{\mathrm{op}})$
$\sqrt[4]{B_0}$	0.023 (0.015, 0.031)	-0.002 (-0.028, 0.024)	0.092 (-8.553, 8.958)	0.002 (-0.031, 0.036)	0.000 (-0.024, 0.025)	0.001 (-0.019, 0.021)
$\ln(E)$		0.323 (0.097, 0.612)	-19.242 (-91.345, 50.098)	-0.037 (-0.263, 0.190)	0.011 (-0.204, 0.227)	-0.121 (-0.305, 0.026)
T_{pk}^2			37610.300 (18344.050, 60589.390)	46.729 (-18.112, 115.761)	18.825 (-56.629, 93.119)	-1.325 (-57.137, 56.747)
$\ln(B_{\mathrm{pk}})$				0.532 (0.268, 0.838)	0.038 (-0.213, 0.293)	0.016 (-0.171, 0.206)
$\ln(E_{\mathrm{D}})$					0.296 (0.086, 0.573)	-0.020 (-0.181, 0.130)
$\ln(W_{\mathrm{op}})$						0.196 (0.066, 0.360)

Table S8: Phylogenetically heritable variance/covariance matrix of TPC parameters, as estimated from the best fitting model (i.e., with absolute latitude as fixed effect).

	$\sqrt[4]{B_0}$	$\ln(E)$	T_{pk}^2	$\ln(B_{\mathrm{pk}})$	$\ln(E_{\mathrm{D}})$	$\ln(W_{\mathrm{op}})$
$\sqrt[4]{B_0}$	0.010 (0.007, 0.012)	-0.002 (-0.012, 0.009)	0.002 (-0.120, 0.122)	0.001 (-0.009, 0.011)	0.000 (-0.007, 0.008)	0.001 (-0.006, 0.007)
$\ln(E)$		0.294 (0.185, 0.408)	-0.199 (-3.237, 2.837)	-0.027 (-0.149, 0.100)	-0.055 (-0.173, 0.058)	-0.094 (-0.184, -0.009)
T_{pk}^2			68.851 (0.066, 216.891)	0.184 (-2.036, 2.654)	0.032 (-1.647, 1.597)	0.072 (-1.378, 1.564)
$\ln(B_{\mathrm{pk}})$				0.237 (0.170, 0.308)	0.009 (-0.083, 0.102)	0.001 (-0.085, 0.087)
$\ln(E_{\mathrm{D}})$					0.149 (0.063, 0.255)	0.015 (-0.049, 0.084)
$\ln(W_{\mathrm{op}})$						0.115 (0.050, 0.191)

Table S9: Residual variance/covariance matrix of TPC parameters, as estimated from the best fitting model (i.e., with absolute latitude as fixed effect).

S5.2 Using the marine subset of the dataset

S5.2.1 With $T_{\mathbf{ref}}$ set to 0°C

Table S10 shows the mean DIC value (averaged across two chains per model) of MCMCglmms fitted on the dataset of marine TPC parameters. Note that a model is included in this table only if each of its fixed effects formed a statistical association with at least one parameter of the TPC (i.e., with at least one response variable). This means that a model was accepted if each of its fixed effects had at least one coefficient whose 95% Highest Posterior Density interval did not include zero. Note that, other than the fixed effects shown in this table, all models also included a distinct intercept for each response variable. The model with the lowest DIC is shown in bold.

Table S10: DIC values for models fitted on the marine subset of the dataset, using a T_{ref} of 0°C.

**Fixed effects**	**Mean DIC**
-	283.16
$\lvert L_{\text{orig}}\rvert$	156.99
$L_{\text{orig}} + L^2_{\text{orig}}$	220.16
$\tilde{T}_{\text{orig}}$	179.97
$\tilde{T}_{\text{orig}} + \text{IQR}(T_{\text{orig}}) + \text{IQR}(T_{\text{orig}})^2$	209.81
$\tilde{T}_{\text{2.5m, 50d}}$	174.82
$\lvert\tilde{L}_{\text{2.5m, 50d}}\rvert$	165.89
$\tilde{L}_{\text{2.5m, 50d}} + \tilde{L}^2_{\text{2.5m, 50d}}$	191.28
$\tilde{T}_{\text{2.5m, 150d}}$	164.03
$\lvert\tilde{L}_{\text{2.5m, 150d}}\rvert$	167.12
$\tilde{L}_{\text{2.5m, 150d}} + \tilde{L}^2_{\text{2.5m, 150d}}$	189.77
$\tilde{T}_{\text{2.5m, 250d}}$	159.90
$\text{IQR}(T_{\text{2.5m, 250d}}) + \text{IQR}(T_{\text{2.5m, 250d}})^2$	275.78
$\lvert\tilde{L}_{\text{2.5m, 250d}}\rvert$	176.21
$\tilde{L}_{\text{2.5m, 250d}} + \tilde{L}^2_{\text{2.5m, 250d}}$	194.92
$\tilde{T}_{\text{2.5m, 350d}}$	161.07
$\text{IQR}(T_{\text{2.5m, 350d}}) + \text{IQR}(T_{\text{2.5m, 350d}})^2$	272.33
$\tilde{T}_{\text{2.5m, 350d}} + \text{IQR}(T_{\text{2.5m, 350d}})$	151.81
$\lvert\tilde{L}_{\text{2.5m, 350d}}\rvert$	173.60
$\tilde{L}_{\text{2.5m, 350d}} + \tilde{L}^2_{\text{2.5m, 350d}}$	205.54
$\tilde{T}_{\text{2.5m, 500d}}$	178.07
$\text{IQR}(T_{\text{2.5m, 500d}}) + \text{IQR}(T_{\text{2.5m, 500d}})^2$	269.53
$\tilde{T}_{\text{2.5m, 500d}} + \text{IQR}(T_{\text{2.5m, 500d}})$	158.44
$\lvert\tilde{L}_{\text{2.5m, 500d}}\rvert$	168.06
$\tilde{L}_{\text{2.5m, 500d}} + \tilde{L}^2_{\text{2.5m, 500d}}$	200.88
$\text{IQR}(T_{\text{50m, 50d}})$	269.99
$\tilde{T}_{\text{50m, 50d}}$	112.37
$\tilde{T}_{\text{50m, 50d}} + \text{IQR}(T_{\text{50m, 50d}})$	121.33
$\lvert\tilde{L}_{\text{50m, 50d}}\rvert$	181.28
$\tilde{L}_{\text{50m, 50d}} + \tilde{L}^2_{\text{50m, 50d}}$	183.27
$\tilde{T}_{\text{50m, 150d}}$	112.04

Table S10 – *Continued from previous page*

Fixed effects	Mean DIC
$\lvert\tilde{L}_{\text{50m, 150d}}\rvert$	174.00
$\tilde{L}_{\text{50m, 150d}} + \tilde{L}^2_{\text{50m, 150d}}$	193.19
$\boldsymbol{\tilde{T}_{\text{50m, 250d}}}$	**110.89**
$\lvert\tilde{L}_{\text{50m, 250d}}\rvert$	171.43
$\tilde{L}_{\text{50m, 250d}} + \tilde{L}^2_{\text{50m, 250d}}$	192.37
$\tilde{T}_{\text{50m, 350d}}$	116.82
$\lvert\tilde{L}_{\text{50m, 350d}}\rvert$	178.80
$\tilde{L}_{\text{50m, 350d}} + \tilde{L}^2_{\text{50m, 350d}}$	186.09
$\tilde{T}_{\text{50m, 500d}}$	116.01
$\lvert\tilde{L}_{\text{50m, 500d}}\rvert$	174.85
$\tilde{L}_{\text{50m, 500d}} + \tilde{L}^2_{\text{50m, 500d}}$	189.50
$\tilde{T}_{\text{100m, 50d}}$	202.33
$\lvert\tilde{L}_{\text{100m, 50d}}\rvert$	176.64
$\tilde{L}_{\text{100m, 50d}} + \tilde{L}^2_{\text{100m, 50d}}$	189.64
$\tilde{T}_{\text{100m, 150d}}$	195.92
$\lvert\tilde{L}_{\text{100m, 150d}}\rvert$	169.88
$\tilde{L}_{\text{100m, 150d}} + \tilde{L}^2_{\text{100m, 150d}}$	202.98
$\tilde{T}_{\text{100m, 250d}}$	198.63
$\lvert\tilde{L}_{\text{100m, 250d}}\rvert$	167.11
$\tilde{L}_{\text{100m, 250d}} + \tilde{L}^2_{\text{100m, 250d}}$	188.91
$\tilde{T}_{\text{100m, 350d}}$	202.08
$\lvert\tilde{L}_{\text{100m, 350d}}\rvert$	172.11
$\lvert\tilde{L}_{\text{100m, 350d}}\rvert + \text{IQR}(L_{\text{100m, 350d}})$	178.78
$\tilde{L}_{\text{100m, 350d}} + \tilde{L}^2_{\text{100m, 350d}}$	193.64
$\tilde{T}_{\text{100m, 500d}}$	189.05
$\lvert\tilde{L}_{\text{100m, 500d}}\rvert$	172.06
$\lvert\tilde{L}_{\text{100m, 500d}}\rvert + \text{IQR}(L_{\text{100m, 500d}})$	169.22
$\tilde{L}_{\text{100m, 500d}} + \tilde{L}^2_{\text{100m, 500d}}$	194.30

The fixed effects of the model with the lowest DIC included i) a distinct intercept for each response variable and ii) $\tilde{T}_{\text{50m, 250d}}$, which we estimated using the simulated Lagrangian trajectories. If we exclude models with variables from drifting trajectories, the best-fitting model had $\lvert L_{\text{orig}}\rvert$ – other than the intercepts – as a fixed effect.

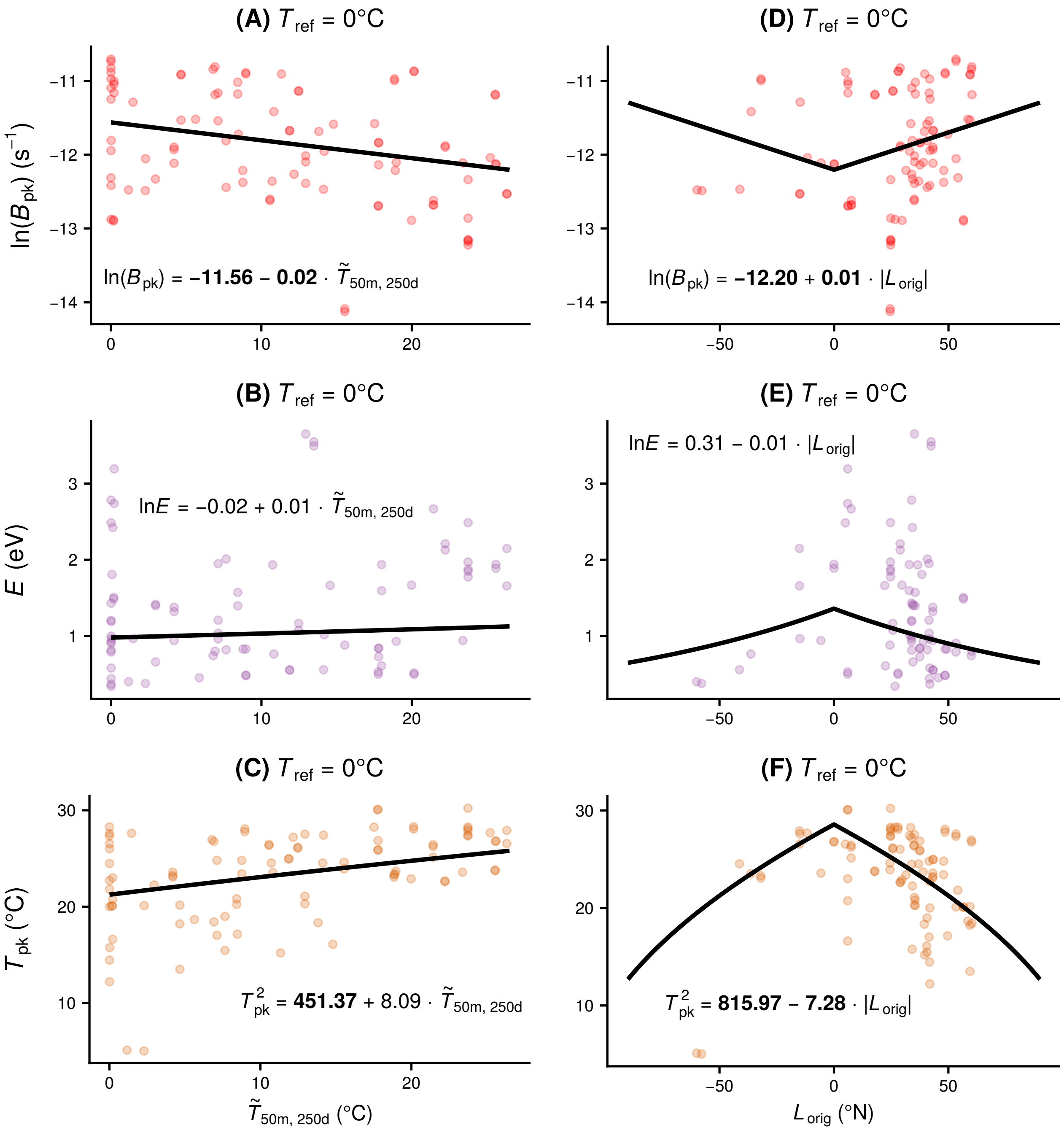

Figure S20: Inferred associations between TPC parameters of marine species/strains and environmental variables. Panels A-C show the relationships of three thermal parameters with the weighted median temperature of estimated drifting trajectories at a depth of 50 meters over 250 days. The MCMCglmm from which the coefficients were obtained for A-C had the lowest DIC among all fitted models. B_{pk} is the only parameter whose $\tilde{T}_{50m,\ 250d}$ coefficient had a 95% HPD interval that did not include zero, and is found to decrease with the temperature of the trajectory. This suggests the presence of cold adaptation in marine phytoplankton, with regard to the maximum height of the thermal performance curve. Panels D-F show the inferred relationships of the same parameters using a model with absolute latitude as fixed effect. Here, both B_{pk} and T_{pk} scale with latitude, with the former increasing and the latter decreasing. E does not scale with an environmental variable in any of the two models.

	$\sqrt[4]{B_0}$	$\ln(E)$	T_{pk}^2	$\ln(B_{\mathrm{pk}})$	$\ln(E_{\mathrm{D}})$	$\ln(W_{\mathrm{op}})$
$\sqrt[4]{B_0}$	0.053 (0.027, 0.084)	-0.004 (-0.074, 0.063)	-0.450 (-24.411, 22.956)	0.002 (-0.068, 0.072)	0.000 (-0.081, 0.080)	0.002 (-0.053, 0.060)
$\ln(E)$		0.425 (0.116, 0.819)	21.369 (-86.149, 133.125)	-0.060 (-0.349, 0.198)	0.007 (-0.367, 0.395)	-0.152 (-0.427, 0.071)
T_{pk}^2			49591.680 (0.111, 87420.630)	38.675 (-52.926, 136.351)	26.886 (-167.099, 230.387)	-16.593 (-108.272, 75.629)
$\ln(B_{\mathrm{pk}})$				0.501 (0.197, 0.875)	0.091 (-0.327, 0.557)	0.045 (-0.183, 0.287)
$\ln(E_{\mathrm{D}})$					0.574 (0.084, 1.430)	-0.003 (-0.323, 0.309)
$\ln(W_{\mathrm{op}})$						0.285 (0.079, 0.574)

Table S11: Phylogenetically heritable variance/covariance matrix of TPC parameters, as estimated from the best fitting model (i.e., with $\tilde{T}_{\mathrm{50m,\ 250d}}$ as fixed effect).

	$\sqrt[4]{B_0}$	$\ln(E)$	T_{pk}^2	$\ln(B_{\mathrm{pk}})$	$\ln(E_{\mathrm{D}})$	$\ln(W_{\mathrm{op}})$
$\sqrt[4]{B_0}$	0.018 (0.012, 0.025)	-0.002 (-0.019, 0.015)	0.012 (-0.699, 0.746)	0.000 (-0.022, 0.022)	0.000 (-0.018, 0.019)	0.001 (-0.013, 0.014)
$\ln(E)$		0.215 (0.113, 0.330)	-0.935 (-9.057, 5.939)	0.000 (-0.158, 0.157)	-0.047 (-0.188, 0.089)	-0.056 (-0.147, 0.030)
T_{pk}^2			1244.907 (0.060, 5824.667)	1.910 (-6.487, 14.600)	0.908 (-6.949, 10.024)	0.521 (-3.842, 5.712)
$\ln(B_{\mathrm{pk}})$				0.268 (0.170, 0.376)	0.042 (-0.144, 0.233)	0.016 (-0.109, 0.138)
$\ln(E_{\mathrm{D}})$					0.249 (0.073, 0.487)	0.013 (-0.088, 0.119)
$\ln(W_{\mathrm{op}})$						0.127 (0.051, 0.223)

Table S12: Residual variance/covariance matrix of TPC parameters, as estimated from the best fitting model (i.e., with $\tilde{T}_{\mathrm{50m,\ 250d}}$ as fixed effect).

	$\sqrt[4]{B_0}$	$\ln(E)$	T_{pk}^2	$\ln(B_{\mathrm{pk}})$	$\ln(E_{\mathrm{D}})$	$\ln(W_{\mathrm{op}})$
$\sqrt[4]{B_0}$	0.053<br>(0.027, 0.084)	-0.004<br>(-0.072, 0.064)	-0.274<br>(-18.690, 17.893)	0.002<br>(-0.067, 0.073)	0.001<br>(-0.078, 0.079)	0.002<br>(-0.055, 0.058)
$\ln(E)$		0.431<br>(0.124, 0.812)	25.421<br>(-57.966, 111.510)	-0.089<br>(-0.371, 0.175)	-0.082<br>(-0.470, 0.268)	-0.160<br>(-0.429, 0.060)
T_{pk}^2			31228.890<br>(8992.526, 66348.630)	30.729<br>(-41.566, 109.598)	-19.051<br>(-158.646, 120.711)	-19.975<br>(-92.052, 49.631)
$\ln(B_{\mathrm{pk}})$				0.489<br>(0.179, 0.858)	0.088<br>(-0.352, 0.564)	0.068<br>(-0.161, 0.311)
$\ln(E_{\mathrm{D}})$					0.562<br>(0.090, 1.336)	0.050<br>(-0.245, 0.368)
$\ln(W_{\mathrm{op}})$						0.291<br>(0.085, 0.577)

Table S13: Phylogenetically heritable variance/covariance matrix of TPC parameters, as estimated from the model with $|L_{\mathrm{orig}}|$ as fixed effect.

	$\sqrt[4]{B_0}$	$\ln(E)$	T_{pk}^2	$\ln(B_{\mathrm{pk}})$	$\ln(E_{\mathrm{D}})$	$\ln(W_{\mathrm{op}})$
$\sqrt[4]{B_0}$	0.018<br>(0.012, 0.025)	-0.002<br>(-0.018, 0.015)	0.003<br>(-0.368, 0.396)	0.000<br>(-0.022, 0.022)	0.000<br>(-0.018, 0.019)	0.001<br>(-0.013, 0.014)
$\ln(E)$		0.202<br>(0.109, 0.313)	-0.379<br>(-4.059, 3.256)	0.003<br>(-0.158, 0.168)	-0.033<br>(-0.172, 0.096)	-0.055<br>(-0.146, 0.026)
T_{pk}^2			450.626<br>(0.061, 765.640)	0.996<br>(-4.219, 6.134)	0.234<br>(-3.928, 4.494)	0.217<br>(-2.294, 2.667)
$\ln(B_{\mathrm{pk}})$				0.283<br>(0.183, 0.395)	0.042<br>(-0.147, 0.245)	0.015<br>(-0.115, 0.142)
$\ln(E_{\mathrm{D}})$					0.235<br>(0.066, 0.469)	0.012<br>(-0.085, 0.120)
$\ln(W_{\mathrm{op}})$						0.127<br>(0.052, 0.223)

Table S14: Residual variance/covariance matrix of TPC parameters, as estimated from the model with $|L_{\mathrm{orig}}|$ as fixed effect.

S5.2.2 With T_{ref} set to 10°C

As in the previous section, we fitted MCMCglmms to the marine subset of the dataset of TPC parameters, which we obtained by setting T_{ref} to 10°C.

Table S15: DIC values for models fitted on the marine subset of the dataset, using a T_{ref} of 10°C.

**Fixed effects**	**Mean DIC**
-	163.07
$\lvert L_{\mathrm{orig}}\rvert$	272.19
$L_{\mathrm{orig}} + L_{\mathrm{orig}}^2$	245.28
$\mathrm{IQR}(T_{\mathrm{orig}})$	200.76
$\tilde{T}_{\mathrm{orig}}$	186.71
$\tilde{T}_{\mathrm{orig}} + \mathrm{IQR}(T_{\mathrm{orig}}) + \mathrm{IQR}(T_{\mathrm{orig}})^2$	197.12
$\tilde{T}_{\mathrm{2.5m,\ 50d}}$	236.99
$\lvert\tilde{L}_{\mathrm{2.5m,\ 50d}}\rvert$	239.42
$\tilde{L}_{\mathrm{2.5m,\ 50d}} + \tilde{L}_{\mathrm{2.5m,\ 50d}}^2$	200.29
$\tilde{T}_{\mathrm{2.5m,\ 150d}}$	185.22
$\lvert\tilde{L}_{\mathrm{2.5m,\ 150d}}\rvert$	248.47
$\tilde{L}_{\mathrm{2.5m,\ 150d}} + \tilde{L}_{\mathrm{2.5m,\ 150d}}^2$	208.08
$\mathrm{IQR}(T_{\mathrm{2.5m,\ 250d}})$	138.56
$\mathrm{IQR}(T_{\mathrm{2.5m,\ 250d}}) + \mathrm{IQR}(T_{\mathrm{2.5m,\ 250d}})^2$	155.51
$\tilde{T}_{\mathrm{2.5m,\ 250d}}$	183.60
$\tilde{T}_{\mathrm{2.5m,\ 250d}} + \mathrm{IQR}(T_{\mathrm{2.5m,\ 250d}})$	451.24
$\lvert\tilde{L}_{\mathrm{2.5m,\ 250d}}\rvert$	237.49
$\tilde{L}_{\mathrm{2.5m,\ 250d}} + \tilde{L}_{\mathrm{2.5m,\ 250d}}^2$	216.69
$\mathrm{IQR}(T_{\mathrm{2.5m,\ 350d}})$	145.10
$\mathrm{IQR}(T_{\mathrm{2.5m,\ 350d}}) + \mathrm{IQR}(T_{\mathrm{2.5m,\ 350d}})^2$	148.96
$\tilde{T}_{\mathrm{2.5m,\ 350d}}$	158.83
$\tilde{T}_{\mathrm{2.5m,\ 350d}} + \mathrm{IQR}(T_{\mathrm{2.5m,\ 350d}})$	413.91
$\lvert\tilde{L}_{\mathrm{2.5m,\ 350d}}\rvert$	251.17
$\tilde{L}_{\mathrm{2.5m,\ 350d}} + \tilde{L}_{\mathrm{2.5m,\ 350d}}^2$	218.70
$\mathrm{IQR}(T_{\mathrm{2.5m,\ 500d}})$	138.05
$\mathrm{IQR}(T_{\mathrm{2.5m,\ 500d}}) + \mathrm{IQR}(T_{\mathrm{2.5m,\ 500d}})^2$	144.98
$\tilde{T}_{\mathrm{2.5m,\ 500d}}$	143.74
$\tilde{T}_{\mathrm{2.5m,\ 500d}} + \mathrm{IQR}(T_{\mathrm{2.5m,\ 500d}})$	359.78
$\lvert\tilde{L}_{\mathrm{2.5m,\ 500d}}\rvert$	246.65
$\tilde{L}_{\mathrm{2.5m,\ 500d}} + \tilde{L}_{\mathrm{2.5m,\ 500d}}^2$	218.54
**$\mathrm{IQR}(T_{\mathrm{50m,\ 50d}})$**	**131.89**
$\tilde{T}_{\mathrm{50m,\ 50d}}$	302.47
$\lvert\tilde{L}_{\mathrm{50m,\ 50d}}\rvert$	238.86
$\tilde{L}_{\mathrm{50m,\ 50d}} + \tilde{L}_{\mathrm{50m,\ 50d}}^2$	216.09
$\tilde{T}_{\mathrm{50m,\ 150d}}$	318.11
$\lvert\tilde{L}_{\mathrm{50m,\ 150d}}\rvert$	244.82
$\tilde{L}_{\mathrm{50m,\ 150d}} + \tilde{L}_{\mathrm{50m,\ 150d}}^2$	207.26

Table S15 – *Continued from previous page*

**Fixed effects**	**Mean DIC**
$\tilde{T}_{\text{50m, 250d}}$	386.06
$\lvert\tilde{L}_{\text{50m, 250d}}\rvert$	234.94
$\tilde{L}_{\text{50m, 250d}} + \tilde{L}^2_{\text{50m, 250d}}$	212.39
$\tilde{T}_{\text{50m, 350d}}$	380.76
$\lvert\tilde{L}_{\text{50m, 350d}}\rvert$	260.08
$\tilde{L}_{\text{50m, 350d}} + \tilde{L}^2_{\text{50m, 350d}}$	208.43
$\tilde{T}_{\text{50m, 500d}}$	396.75
$\lvert\tilde{L}_{\text{50m, 500d}}\rvert$	239.78
$\tilde{L}_{\text{50m, 500d}} + \tilde{L}^2_{\text{50m, 500d}}$	212.37
$\tilde{T}_{\text{100m, 50d}}$	145.56
$\lvert\tilde{L}_{\text{100m, 50d}}\rvert$	246.17
$\tilde{L}_{\text{100m, 50d}} + \tilde{L}^2_{\text{100m, 50d}}$	215.50
$\tilde{T}_{\text{100m, 150d}}$	150.16
$\lvert\tilde{L}_{\text{100m, 150d}}\rvert$	245.48
$\tilde{L}_{\text{100m, 150d}} + \tilde{L}^2_{\text{100m, 150d}}$	223.21
$\tilde{T}_{\text{100m, 250d}}$	153.08
$\lvert\tilde{L}_{\text{100m, 250d}}\rvert$	255.41
$\tilde{L}_{\text{100m, 250d}} + \tilde{L}^2_{\text{100m, 250d}}$	223.60
$\tilde{T}_{\text{100m, 350d}}$	146.88
$\lvert\tilde{L}_{\text{100m, 350d}}\rvert$	244.97
$\tilde{L}_{\text{100m, 350d}} + \tilde{L}^2_{\text{100m, 350d}}$	217.77
$\tilde{T}_{\text{100m, 500d}}$	144.93
$\lvert\tilde{L}_{\text{100m, 500d}}\rvert$	244.13
$\tilde{L}_{\text{100m, 500d}} + \tilde{L}^2_{\text{100m, 500d}}$	208.25

The fixed effects of the best-fitting model overall included i) a distinct intercept for each response variable and ii) IQR($T_{\text{50m, 50d}}$), which was obtained from the simulated Lagrangian trajectories. If we were to exclude models with Lagrangian trajectory variables, the model with the lowest DIC was the intercepts-only model.

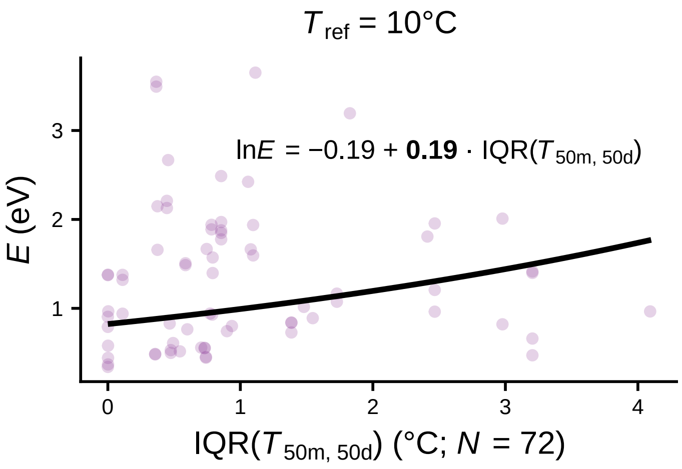

Figure S21: A positive scaling of E with IQR($T_{50m,\ 50d}$) was detected for the marine subset of the dataset and using a T_{ref} of 10°C.

	$\sqrt[4]{B_0}$	$\ln(E)$	T_{pk}^2	$\ln(B_{\mathrm{pk}})$	$\ln(E_{\mathrm{D}})$	$\ln(W_{\mathrm{op}})$
$\sqrt[4]{B_0}$	0.058<br>(0.029, 0.093)	-0.004<br>(-0.081, 0.072)	-0.591<br>(-25.208, 25.056)	0.004<br>(-0.072, 0.076)	0.000<br>(-0.080, 0.080)	0.002<br>(-0.060, 0.065)
$\ln(E)$		0.451<br>(0.135, 0.856)	2.570<br>(-114.448, 121.005)	-0.147<br>(-0.449, 0.111)	0.004<br>(-0.364, 0.383)	-0.163<br>(-0.448, 0.070)
T_{pk}^2			47998.560<br>(15406.420, 101940.100)	-24.506<br>(-123.608, 73.765)	13.669<br>(-142.435, 171.837)	-9.701<br>(-108.015, 84.478)
$\ln(B_{\mathrm{pk}})$				0.472<br>(0.179, 0.834)	-0.014<br>(-0.412, 0.387)	0.088<br>(-0.132, 0.337)
$\ln(E_{\mathrm{D}})$					0.492<br>(0.084, 1.164)	-0.007<br>(-0.314, 0.296)
$\ln(W_{\mathrm{op}})$						0.300<br>(0.082, 0.594)

Table S16: Phylogenetically heritable variance/covariance matrix of TPC parameters, as estimated from the best fitting model (i.e., with IQR($T_{\mathrm{50m,\ 50d}}$) as fixed effect).

	$\sqrt[4]{B_0}$	$\ln(E)$	T_{pk}^2	$\ln(B_{\mathrm{pk}})$	$\ln(E_{\mathrm{D}})$	$\ln(W_{\mathrm{op}})$
$\sqrt[4]{B_0}$	0.021<br>(0.014, 0.030)	-0.001<br>(-0.021, 0.018)	0.007<br>(-0.516, 0.574)	0.001<br>(-0.028, 0.029)	0.000<br>(-0.021, 0.023)	0.000<br>(-0.015, 0.016)
$\ln(E)$		0.205<br>(0.104, 0.325)	-1.078<br>(-5.989, 3.946)	-0.039<br>(-0.203, 0.123)	-0.055<br>(-0.198, 0.074)	-0.051<br>(-0.142, 0.033)
T_{pk}^2			786.781<br>(0.067, 1209.402)	0.501<br>(-5.385, 7.145)	0.514<br>(-4.720, 6.011)	0.439<br>(-2.933, 3.418)
$\ln(B_{\mathrm{pk}})$				0.320<br>(0.202, 0.450)	0.035<br>(-0.196, 0.273)	0.016<br>(-0.125, 0.159)
$\ln(E_{\mathrm{D}})$					0.256<br>(0.070, 0.515)	0.014<br>(-0.093, 0.125)
$\ln(W_{\mathrm{op}})$						0.128<br>(0.050, 0.224)

Table S17: Residual variance/covariance matrix of TPC parameters, as estimated from the best fitting model (i.e., with IQR($T_{\mathrm{50m,\ 50d}}$) as fixed effect).

	$\sqrt[4]{B_0}$	$\ln(E)$	T_{pk}^2	$\ln(B_{\text{pk}})$	$\ln(E_{\text{D}})$	$\ln(W_{\text{op}})$
$\sqrt[4]{B_0}$	0.057<br>(0.029, 0.093)	-0.003<br>(-0.081, 0.073)	-0.554<br>(-25.419, 24.524)	0.003<br>(-0.068, 0.076)	0.000<br>(-0.078, 0.077)	0.002<br>(-0.059, 0.062)
$\ln(E)$		0.457<br>(0.122, 0.881)	-0.078<br>(-120.612, 115.701)	-0.126<br>(-0.427, 0.137)	-0.009<br>(-0.373, 0.373)	-0.158<br>(-0.446, 0.075)
T_{pk}^2			49565.850<br>(17809.710, 93889.040)	-21.044<br>(-119.439, 76.093)	-6.786<br>(-152.725, 141.140)	-10.779<br>(-104.098, 84.569)
$\ln(B_{\text{pk}})$				0.455<br>(0.170, 0.815)	-0.054<br>(-0.305, 0.423)	0.080<br>(-0.138, 0.321)
$\ln(E_{\text{D}})$					0.469<br>(0.080, 1.088)	0.008<br>(-0.283, 0.295)
$\ln(W_{\text{op}})$						0.291<br>(0.080, 0.578)

Table S18: Phylogenetically heritable variance/covariance matrix of TPC parameters, as estimated from the intercepts-only model.

	$\sqrt[4]{B_0}$	$\ln(E)$	T_{pk}^2	$\ln(B_{\text{pk}})$	$\ln(E_{\text{D}})$	$\ln(W_{\text{op}})$
$\sqrt[4]{B_0}$	0.021<br>(0.014, 0.029)	-0.001<br>(-0.022, 0.019)	0.002<br>(-0.378, 0.387)	0.001<br>(-0.028, 0.029)	0.001<br>(-0.023, 0.024)	0.000<br>(-0.015, 0.016)
$\ln(E)$		0.231<br>(0.119, 0.367)	-0.653<br>(-4.550, 3.190)	-0.052<br>(-0.241, 0.151)	-0.070<br>(-0.238, 0.086)	-0.060<br>(-0.165, 0.032)
T_{pk}^2			412.268<br>(0.065, 528.633)	0.481<br>(-4.301, 5.433)	0.371<br>(-3.861, 4.949)	0.279<br>(-1.980, 2.533)
$\ln(B_{\text{pk}})$				0.328<br>(0.209, 0.462)	0.084<br>(-0.147, 0.322)	0.023<br>(-0.125, 0.170)
$\ln(E_{\text{D}})$					0.288<br>(0.085, 0.575)	0.018<br>(-0.101, 0.137)
$\ln(W_{\text{op}})$						0.130<br>(0.050, 0.230)

Table S19: Residual variance/covariance matrix of TPC parameters, as estimated from the intercepts-only model.

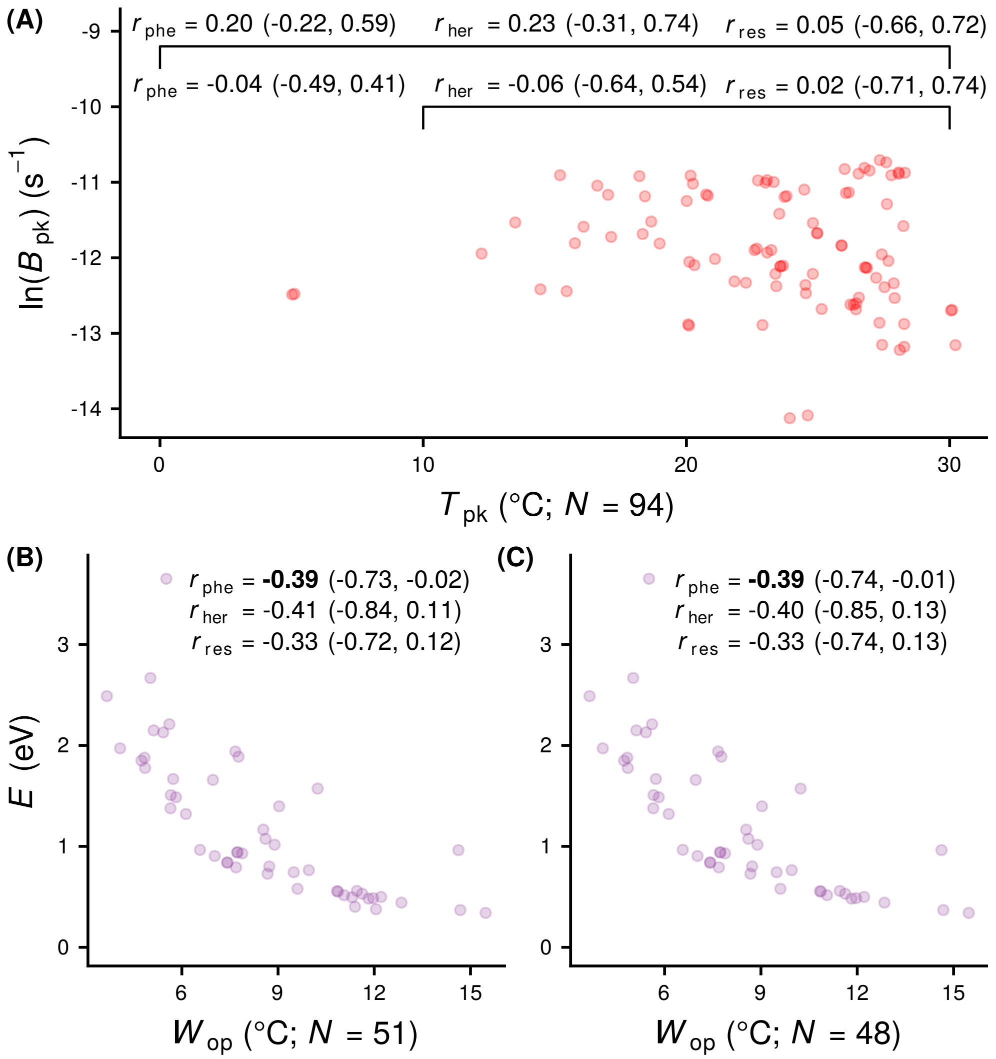

Figure S22: Inferred correlations between TPC parameters of marine species/strains. A: A positive correlation between T_{pk} and B_{pk} – as suggested by the "hotter-is-better" hypothesis – could not be detected. This is partly expected from figure S20, as B_{pk} increases with absolute latitude, whereas T_{pk} decreases. B-C: A correlation between $\ln(E)$ and $\ln(W_{\text{op}})$ can still be detected regardless of T_{ref} value (0°C in panel B, 10°C in C), albeit marginally, possibly due to the smaller sample size.

S6 Scaling of B_0 and B_{pk} with cell volume

S6.1 Regressions with B_0 and B_{pk} appropriately transformed towards normality

To detect any effects of cell volume on growth rate at low temperatures (B_0) or on the maximum possible growth rate for each TPC (B_{pk}), we performed the regressions shown in Table S20 using the MCMCglmm R package. The models with the lowest DIC for each response variable are shown in Fig. 5 in the main text.

Table S20: DIC values for regressions of $\sqrt[4]{B_0}$ or $\ln(B_{pk})$ against the natural logarithm of cell volume.

**Model structure**	**Response variable**			
	$T_{ref} = 0°C$		$T_{ref} = 10°C$	
	$\sqrt[4]{B_0}$	$\ln(B_{pk})$	$\sqrt[4]{B_0}$	$\ln(B_{pk})$
Species identity as a random effect on the intercept	-473.50	**316.82**	-424.18	**314.76**
Species identity as a random effect on the intercept, phylogenetic correction	**-518.06**	328.00	**-467.62**	327.02
Species identity as a random effect on the slope	-388.56	329.40	-336.52	326.43
Species identity as a random effect on the slope, phylogenetic correction	-389.57	328.22	-337.66	325.46
Species identity as a random effect on the intercept and the slope	-388.55	329.55	-336.50	326.60
Species identity as a random effect on the intercept and the slope, phylogenetic correction	-389.42	328.32	-337.64	325.49

S6.2 Regressions with log-transformed B_0

Table S21: DIC values for regressions of $\ln(B_0)$ against the natural logarithm of cell volume.

**Model structure**	T_{ref}	
	**0°C**	**10°C**
Species identity as a random effect on the intercept	**786.65**	**440.46**
Species identity as a random effect on the intercept, phylogenetic correction	811.78	493.22
Species identity as a random effect on the slope	790.50	444.88
Species identity as a random effect on the slope, phylogenetic correction	795.21	450.95
Species identity as a random effect on the intercept and the slope	790.78	445.04
Species identity as a random effect on the intercept and the slope, phylogenetic correction	795.30	450.66

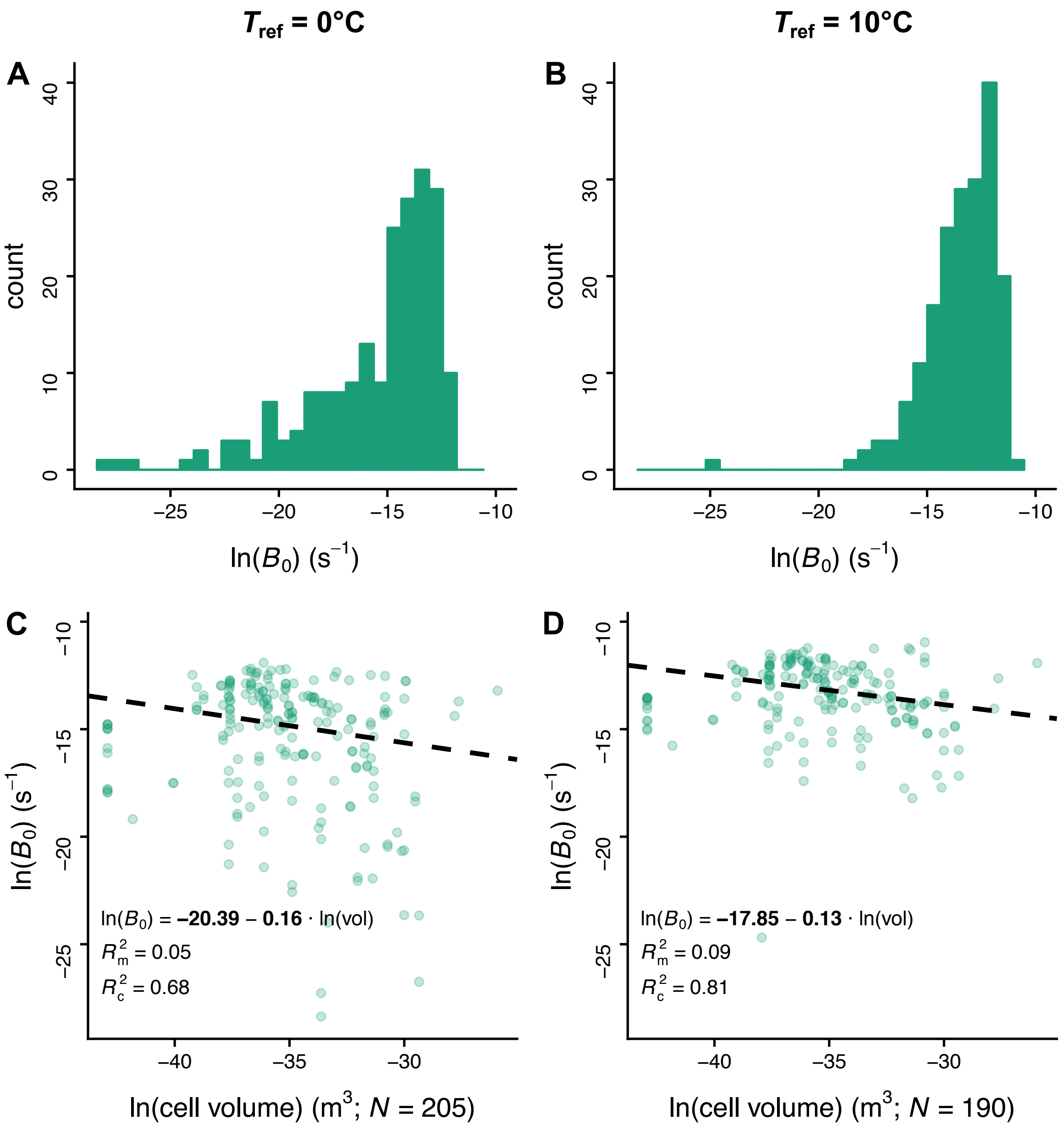

Figure S23: Scaling of $\ln(B_0)$ with the natural logarithm of cell volume. For both T_{ref} values, the distribution of $\ln(B_0)$ estimates deviates strongly from the Gaussian (panels A, B), violating the normality assumption of the fitted MCMCglmms. It is worth noting, however, that the distribution is more similar to the Gaussian at $T_{\mathrm{ref}} = 10°\mathrm{C}$ than at 0°C. The amount of variance that is explained by the natural logarithm of cell volume (R^2_{m}; panels C, D) is very small in both cases but increases with T_{ref}. These results, along with the slightly stronger scaling of B_{pk} with cell volume (Fig. 5 in the main text), indicate that cell volume mainly influences rate performance near the peak of the TPC and not at low temperatures.

S7 List of 16S/18S rRNA sequences used for phylogeny reconstruction

Table S22: Species names and Accession IDs of sequences that were used in this study.

**Species**	**Accession ID**
Abies alba	GenBank: DQ371809.1
Abutilon theophrasti	GenBank: DQ287985.1
Acer rubrum	GenBank: U42494.1
Acutodesmus obliquus	GenBank: KC852905.1
Aldrovanda vesiculosa	GenBank: AY096114.1
Alexandrium catenella	GenBank: AJ535392.1
Alexandrium fundyense	GenBank: KF908796.1
Alexandrium minutum	GenBank: U27499.1
Alexandrium monilatum	GenBank: AY883005.1
Alexandrium ostenfeldii	GenBank: U27500.1
Alexandrium tamarense	GenBank: AJ415510.1
Alexandrium tamiyavanichi	GenBank: AB088325.1
Amphidinium klebsii	GenBank: EU046335.1
Amphiprora paludosa	GenBank: AY485468.1
Anabaena bergii	GenBank: AF160256.1
Anabaena cylindrica	GenBank: HF678516.1
Anabaena macrospora	GenBank: AJ293115.1
Anabaena spiroides	GenBank: AB271212.1
Anabaena ucrainica	GenBank: AB551452.1
Ankistrodesmus falcatus var. *tumidus*	GenBank: JQ315498.1
Apedinella radians	GenBank: U14384.1
Aphanizomenon gracile	GenBank: AJ293127.1
Aphanizomenon ovalisporum	GenBank: FM177484.1
Aplectrum hyemale	GenBank: U59937.1
Arbutus unedo	GenBank: AF206853.1
Aristotelia serrata	GenBank: GU476422.1
Arthrospira fusiformis	GenBank: AF260510.2
Asterionella formosa	GenBank: AM712617.1
Asterionellopsis glacialis	GenBank: X77701.1
Aulacoseira baicalensis	GenBank: AY121821.1
Aulacoseira granulata	GenBank: AB430586.1
Aulacoseira subarctica	GenBank: AY121818.1
Betula papyrifera	GenBank: L00971.1
Betula pendula	GenBank: GU476453.1
Botryococcus braunii	GenBank: AB780365.1
Brassica oleracea	GenBank: KJ607174.1
Brassica rapa	GenBank: LC009534.1
Bryum argenteum	GenBank: U18529.1
Capsicum annuum	GenBank: EF564281.1

Table S22 – *Continued from previous page*

Species	Accession ID
Carya glabra	GenBank: AF206880.1
Caulerpa serrulata	GenBank: JQ745683.1
Ceratium furca	GenBank: AJ276699.1
Ceratium furcoides	GenBank: JQ639757.1
Ceratium fusus	GenBank: AF022153.1
Ceratophyllum demersum	GenBank: U42517.1
Chaetoceros calcitrans	GenBank: JF489975.1
Chaetoceros debilis	GenBank: AB847419.1
Chaetoceros didymus	GenBank: X85392.2
Chaetoceros gracilis	GenBank: AY625895.1
Chamaebatiaria millefolium	GenBank: DQ886366.1
Chatonella marina	GenBank: AB217627.1
Chlamydomonas acidophila	GenBank: AJ852427.1
Chlamydomonas globosa	GenBank: AB753039.1
Chlamydomonas reinhardtii	GenBank: KF864473.1
Chlamydomonas subcaudata	GenBank: AJ781310.1
Chlorella ellipsoidea	GenBank: X63520.1
Chlorella pyrenoidosa	GenBank: AB240151.1
Chlorella saccharophila	GenBank: AB183577.1
Chlorella sorokiniana	GenBank: EU402596.1
Chlorella vulgaris	GenBank: HQ702325.1
Chroococcus minutus	GenBank: GQ375047.1
Chroomonas salina	GenBank: GU983864.1
Chrysanthemum morifolium	GenBank: KJ870235.1
Chrysochromulina acantha	GenBank: AJ246278.1
Chrysochromulina simplex	GenBank: AM491021.2
Cicer arietinum	GenBank: AJ011011.4
Citrus aurantium	GenBank: U38312.1
Citrus limon	GenBank: KJ740202.1
Cladophora glomerata	GenBank: AB665579.1
Closterium acerosum	GenBank: AF352230.1
Closterium ehrenbergii	GenBank: AF352228.1
Coccolithus pelagicus	GenBank: AJ246261.1
Cochlodinium polykrikoides	GenBank: EU418971.1
Coelastrum astroideum	GenBank: AF388380.1
Coelastrum microporum	GenBank: JQ315527.1
Coolia monotis	GenBank: EF492487.1
Coscinodiscus concinnus	GenBank: HQ912681.1
Coscinodiscus granii	GenBank: AY485495.1
Coscinodiscus jonesianus	GenBank: KJ577852.1
Coscinodiscus wailesii	GenBank: HQ912668.1
Cosmarium biretum	GenBank: AM920339.1
Cosmarium botrytis	GenBank: AM920378.1
Cosmarium crenatum	GenBank: AM920370.1

Table S22 – *Continued from previous page*

Species	Accession ID
Cosmarium meneghinii	GenBank: AM920366.1
Cosmarium punctulatum	GenBank: AM920373.1
Cosmarium subprotumidum	GenBank: AM920375.1
Crocosphaera watsonii	GenBank: AY620237.1
Cryptomonas curvata	GenBank: JX490049.1
Cryptomonas erosa	GenBank: AM396361.1
Cryptomonas marssonii	GenBank: EU163586.1
Cryptomonas ovata	GenBank: KC928318.1
Cryptomonas pyrenoidifera	GenBank: AJ566180.1
Cucumis sativus	GenBank: AF206894.1
Cyclotella cryptica	GenBank: AY485499.1
Cyclotella meneghiniana	GenBank: HM805030.1
Cylindrospermopsis raciborskii	GenBank: AF516730.1
Cylindrotheca closterium	GenBank: GQ468542.1
Desmidium swartzii	GenBank: AJ428133.1
Desmodesmus abundans	GenBank: KC861671.1
Desmodesmus maximus	GenBank: KJ094574.1
Detonula confervacea	GenBank: HQ912617.1
Diapensia lapponica	GenBank: AF419794.1
Diatoma tenue	GenBank: AJ535143.1
Dinobryon divergens	GenBank: EU025020.1
Ditylum brightwellii	GenBank: X85386.2
Dolichospermum flosaquae	GenBank: AB042858.1
Dunaliella bioculata	GenBank: DQ009761.1
Dunaliella primolecta	GenBank: DQ009764.1
Dunaliella salina	GenBank: DQ447648.1
Dunaliella tertiolecta	GenBank: EF473747.1
Dunaliella viridis	GenBank: DQ009776.1
Egeria densa	GenBank: JF975484.1
Elodea canadensis	GenBank: AF168841.1
Enteromorpha intestinalis	GenBank: AJ000040.1
Eucampia zodiacus	GenBank: KC309495.1
Euglena gracilis	GenBank: AY029409.1
Eutreptiella gymnastica	GenBank: FJ719618.1
Eutreptiella pomquetensis	GenBank: AJ532398.1
Fibrocapsa japonica	GenBank: AY788931.1
Fontinalis antipyretica	GenBank: AF023714.1
Fragilaria barbararum	GenBank: AJ971376.1
Fragilaria bidens	GenBank: AM497732.1
Fragilaria capucina	GenBank: EF465471.1
Fragilaria crotonensis	GenBank: AM712616.1
Fragilariopsis cylindrus	GenBank: EF140624.1
Fragilariopsis kerguelensis	GenBank: KJ866919.1
Gambierdiscus toxicus	GenBank: EF202890.1

Table S22 – *Continued from previous page*

Species	Accession ID
Gephyrocapsa oceanica	GenBank: KC404159.1
Glycine max	GenBank: X02623.1
Gonatozygon monotaenium	GenBank: AJ428084.1
Gonyostomum semen	GenBank: AB512123.1
Gossypium hirsutum	GenBank: L24145.1
Grammonema striatula	GenBank: X77704.1
Guinardia flaccida	GenBank: AJ535191.1
Gymnodinium breve	GenBank: AF172714.1
Gymnodinium catenatum	GenBank: AF022193.1
Gymnodinium mikimotoi	GenBank: AF022195.1
Gymnodinium sanguineum	GenBank: AJ415513.1
Gyrodinium aureolum	GenBank: AF172713.1
Gyrodinium instriatum	GenBank: DQ084522.1
Gyrodinium uncatenum	GenBank: EF492498.1
Haematococcus pluvialis	GenBank: JQ315539.1
Haptolina ericina	GenBank: AM491030.2
Haptolina hirta	GenBank: AJ246272.1
Helianthus annuus	GenBank: AF107577.1
Heterocapsa triquetra	GenBank: AF022198.1
Heterosigma akashiwo	GenBank: AB217869.1
Hordeum vulgare	GenBank: AY552749.1
Hydrilla verticillata	GenBank: KM982363.1
Ipomoea batatas	GenBank: HM053485.1
Isochrysis galbana	GenBank: AJ246266.1
Katodinium rotundatum	GenBank: AF274267.1
Koliella antarctica	GenBank: AJ311569.1
Lactuca sativa	GenBank: M82530.1
Lantana camara	GenBank: AJ236049.1
Larix decidua	GenBank: AB026938.1
Larrea tridentata	GenBank: AY929372.1
Lauderia annulata	GenBank: DQ514849.1
Lemna minor	GenBank: S67398.1
Leptocylindrus danicus	GenBank: AJ535175.1
Leptolyngbya tenuis	GenBank: GQ859652.1
Limnothrix redekei	GenBank: FM177493.1
Lingulodinium polyedrum	GenBank: AB693195.1
Liriodendron tulipifera	GenBank: AF206954.1
Lithophyllum margaritae	GenBank: KP192392.1
Lobochlamys segnis	GenBank: AJ410456.1
Lolium perenne	GenBank: AY519271.1
Mallomonas acaroides	GenBank: JX946333.1
Mallomonas caudata	GenBank: U73228.1
Mallomonas crassisquama	GenBank: KM817866.1
Mallomonas elongata	GenBank: GU935621.1

Table S22 – *Continued from previous page*

**Species**	**Accession ID**
Mallomonas tonsurata	GenBank: GU935620.1
Mastigocladus laminosus	GenBank: DQ431003.1
Merismopedia tenuissima	GenBank: AJ639891.1
Mesotaenium kramstae	GenBank: AJ553922.1
Micractinium pusillum	GenBank: AM231738.1
Micrasterias americana	GenBank: FR852595.1
Microcoleus vaginatus	GenBank: EF654062.1
Microcystis aeruginosa	NCBI Reference Sequence: NR_074314.1
Microcystis ichthyoblabe	GenBank: AJ635433.1
Microcystis viridis	GenBank: U40332.2
Microcystis wesenbergii	GenBank: U40334.1
Micromonas pusilla	GenBank: KP899802.1
Monodus subterranea	GenBank: KF848930.1
Monoraphidium contortum	GenBank: AY846375.1
Monoraphidium convolutum	GenBank: AY846377.1
Monoraphidium griffithii	GenBank: AY846378.1
Mucidosphaerium pulchellum	GenBank: AY323838.1
Mucuna pruriens	GenBank: AF525695.1
Mychonastes homosphaera	GenBank: X73996.1
Nannochloris atomus	GenBank: AB080303.1
Nannochloropsis oceanica	GenBank: FJ896231.1
Nannochloropsis oculata	GenBank: AF045045.1
Navicula arenaria	GenBank: KJ961668.1
Navicula pelliculosa	GenBank: AY485454.1
Nerium oleander	GenBank: AF107572.1
Nicotiana tabacum	GenBank: AJ236016.1
Nitzschia dissipata	GenBank: AJ867018.1
Nitzschia frigida	GenBank: JQ582669.1
Nitzschia paleacea	GenBank: AJ866996.1
Nitzschia sigma	GenBank: AJ867279.1
Nostoc muscorum	GenBank: HF678509.1
Nothofagus grandis	GenBank: GU476452.1
Odontella aurita	GenBank: HQ912687.1
Odontella mobiliensis	GenBank: KC309500.1
Odontella regia	GenBank: KC309502.1
Odontella sinensis	GenBank: HQ912564.1
Olea europaea	GenBank: L49289.1
Olisthodiscus luteus	GenBank: AY788937.1
Oryza sativa	GenBank: AF069218.1
Ostreopsis ovata	GenBank: AF244939.1
Pandorina morum	GenBank: JQ315554.1
Pavlova lutheri	GenBank: AF102369.1
Pediastrum boryanum	GenBank: AY663036.1
Pediastrum duplex	GenBank: M62997.1

Table S22 – *Continued from previous page*

Species	Accession ID
Pelagomonas calceolata	GenBank: EF455763.1
Peridinium bipes	GenBank: DQ372891.1
Phaeocystis antarctica	GenBank: JN381495.1
Phaeocystis globosa	GenBank: AY851301.1
Phaeocystis pouchetii	GenBank: AJ278036.1
Phaeodactylum tricornutum	GenBank: GQ452861.1
Picea mariana	GenBank: L01782.1
Picochlorum oculatum	GenBank: AY422075.1
Pinus elliottii	GenBank: AF051798.1
Planktothrix agardhii	GenBank: FJ159128.1
Planktothrix mougeotii	GenBank: FJ434250.1
Plantago lanceolata	GenBank: AJ236046.1
Pleodorina californica	GenBank: FJ610145.1
Pleurotaenium trabecula	GenBank: AJ428131.1
Populus tremuloides	GenBank: AF206999.1
Porphyridium purpureum	GenBank: AB045584.1
Posidonia australis	GenBank: GQ497582.1
Posidonia oceanica	GenBank: AY491942.1
Potamogeton perfoliatus	GenBank: AY952389.1
Proboscia indica	GenBank: AY485470.1
Proboscia inermis	GenBank: EF192984.1
Prochlorococcus marinus	GenBank: AF180967.1
Prorocentrum concavum	GenBank: Y16237.1
Prorocentrum dentatum	GenBank: AY551273.1
Prorocentrum donghaiense	GenBank: AJ841810.1
Prorocentrum gracile	GenBank: AY443019.1
Prorocentrum lima	GenBank: Y16235.1
Prorocentrum mexicanum	GenBank: Y16232.1
Prorocentrum micans	GenBank: AJ415519.1
Prorocentrum minimum	GenBank: JF715165.1
Prunus persica	GenBank: L28749.1
Prymnesium parvum	GenBank: AJ246269.1
Prymnesium polylepis	GenBank: AJ004866.1
Pseudochattonella farcimen	GenBank: AM075624.1
Pseudochattonella verruculosa	GenBank: AM075625.1
Pseudodidymocystis planctonica	GenBank: AB037087.1
Pseudo-nitzschia granii	GenBank: GU373962.1
Pseudo-nitzschia multiseries	GenBank: AM235382.1
Pseudo-nitzschia pseudodelicatissima	GenBank: GU373965.1
Pseudo-nitzschia seriata	GenBank: GU373969.1
Pycnococcus provasolii	GenBank: X91264.1
Pyramimonas disomata	GenBank: FN562440.1
Pyrodinium bahamense	GenBank: DQ500120.1
Quercus rubra	GenBank: AF132892.1

Table S22 – *Continued from previous page*

Species	Accession ID
Quercus suber	GenBank: GU476438.1
Ranunculus acris	GenBank: M82509.1
Raphidocelis subcapitata	GenBank: HM483520.1
Rhizosolenia robusta	GenBank: AY485481.1
Rhizosolenia setigera	GenBank: AY485461.1
Rhodomonas salina	GenBank: HM126532.1
Rosa hybrida	GenBank: X66773.1
Roya anglica	GenBank: AJ428081.1
Ruppia maritima	GenBank: JN034103.1
Scenedesmus acuminatus	GenBank: AB037088.1
Scenedesmus acutus	GenBank: AJ249512.1
Scenedesmus dimorphus	GenBank: KC790431.1
Scenedesmus quadricauda	GenBank: KC790429.1
Scrippsiella trochoidea	GenBank: EF492513.1
Selenastrum minutum	GenBank: AY846380.1
Skeletonema ardens	GenBank: DQ396522.1
Skeletonema costatum	GenBank: JF489959.1
Skeletonema japonicum	GenBank: DQ011160.1
Skeletonema marinoi	GenBank: JF489953.1
Skeletonema menzelii	GenBank: AJ535168.1
Skeletonema pseudocostatum	GenBank: X85393.1
Skeletonema tropicum	GenBank: EF138941.1
Solanum lycopersicum	GenBank: KJ813722.1
Solanum tuberosum	GenBank: FJ710157.1
Solenostemon scutellarioides	GenBank: EU019244.1
Sorghum bicolor	GenBank: M82328.1
Sphaerospermopsis aphanizomenoides	GenBank: GU197654.1
Sphagnum angustifolium	GenBank: GQ375058.1
Sphagnum squarrosum	GenBank: GQ375075.1
Spinacia oleracea	GenBank: L24420.1
Spirulina platensis	GenBank: AB074508.1
Staurastrum avicula	GenBank: EF507555.1
Staurastrum pingue	GenBank: AJ428109.1
Staurodesmus cuspidatus	GenBank: EF507538.1
Stellarima microtrias	GenBank: EU090011.1
Stephanodiscus hantzschii	GenBank: DQ093370.1
Stephanopyxis palmeriana	GenBank: AY485527.1
Synechococcus elongatus	GenBank: HF678511.1
Synechococcus lividus	GenBank: AF132772.1
Synedra acus	GenBank: AM497723.1
Synura petersenii	GenBank: U73223.1
Synura sphagnicola	GenBank: U73221.1
Syracosphaera pulchra	GenBank: AM490987.2
Thalassionema nitzschioides	GenBank: X77702.2

Table S22 – *Continued from previous page*

Species	Accession ID
Thalassiosira allenii	GenBank: HM991688.1
Thalassiosira curviseriata	GenBank: AJ810859.1
Thalassiosira eccentrica	GenBank: X85396.1
Thalassiosira guillardii	GenBank: AF374478.2
Thalassiosira hendeyi	GenBank: AM050629.1
Thalassiosira nordenskioeldii	GenBank: DQ093365.1
Thalassiosira oceanica	GenBank: DQ093364.1
Thalassiosira pseudonana	GenBank: AY485452.1
Thalassiosira rotula	GenBank: AF374480.2
Thalassiosira weissflogii	GenBank: AY485445.1
Trichodesmium erythraeum	NCBI Reference Sequence: NR_074275.1
Trichormus variabilis	GenBank: DQ234833.1
Triticum aestivum	GenBank: AY049040.1
Tychonema bourrellyi	GenBank: FJ184385.1
Ulva lactuca	GenBank: KF419328.1
Vallisneria americana	GenBank: AF069201.1
Vitis vinifera	GenBank: GQ849399.1
Zea mays	GenBank: AF168884.1
Zostera marina	GenBank: HQ445940.1
Zostera noltii	GenBank: AF207058.1

References

Bogdanowicz, D., and K. Giaro. 2013. On a matching distance between rooted phylogenetic trees. Int. J. Appl. Math. Comput. Sci. 23:669–684.

Bogdanowicz, D., K. Giaro, and B. Wróbel. 2012. TreeCmp: Comparison of trees in polynomial time. Evol. Bioinform. Online 8:475–487.

Dray, S., and A.-B. Dufour. 2007. The ade4 package: implementing the duality diagram for ecologists. J. Stat. Softw. 22:1–20.

Hinchliff, C. E., S. A. Smith, J. F. Allman, J. G. Burleigh, R. Chaudhary, L. M. Coghill, K. A. Crandall, J. Deng, B. T. Drew, R. Gazis, K. Gude, D. S. Hibbett, L. A. Katz, H. D. Laughinghouse, E. J. McTavish, P. E. Midford, C. L. Owen, R. H. Ree, J. A. Rees, D. E. Soltis, T. Williams, and K. A. Cranston. 2015. Synthesis of phylogeny and taxonomy into a comprehensive tree of life. P. Natl. Acad. Sci. USA 112:12764–12769.

Kontopoulos, D. G., B. García-Carreras, S. Sal, T. P. Smith, and S. Pawar. 2018. Use and misuse of temperature normalisation in meta-analyses of thermal responses of biological traits. PeerJ 6:e4363.

Mantel, N. 1967. The detection of disease clustering and a generalized regression approach. Cancer Res. 27:209–220.

Nylander, J. A., J. C. Wilgenbusch, D. L. Warren, and D. L. Swofford. 2007. AWTY (are we there yet?): a system for graphical exploration of MCMC convergence in Bayesian phylogenetics. Bioinformatics 24:581–583.

Pawar, S., A. I. Dell, V. M. Savage, and J. L. Knies. 2016. Real versus artificial variation in the thermal sensitivity of biological traits. Am. Nat. 187:E41–E52.

Schoolfield, R., P. Sharpe, and C. Magnuson. 1981. Non-linear regression of biological temperature-dependent rate models based on absolute reaction-rate theory. J. Theor. Biol. 88:719–731.

Vincenty, T. 1975. Direct and inverse solutions of geodesics on the ellipsoid with application of nested equations. Surv. Rev. 23:88–93.

Warren, D. L., A. J. Geneva, and R. Lanfear. 2017. RWTY (R We There Yet): an R package for examining convergence of Bayesian phylogenetic analyses. Mol. Biol. Evol. 34:1016–1020.

Yu, G., D. Smith, H. Zhu, Y. Guan, and T. T.-Y. Lam. 2017. ggtree: an R package for visualization and annotation of phylogenetic trees with their covariates and other associated data. Methods Ecol. Evol. 8:28–36.
